# Phase I Study of Oral Vinorelbine in Combination with Erlotinib in Advanced Non-Small Cell Lung Cancer (NSCLC) Using Two Different Schedules

**DOI:** 10.1371/journal.pone.0154316

**Published:** 2016-05-02

**Authors:** Natalia Sutiman, Zhenxian Zhang, Eng Huat Tan, Mei Kim Ang, Shao-Weng Daniel Tan, Chee Keong Toh, Quan Sing Ng, Balram Chowbay, Wan-Teck Lim

**Affiliations:** 1 Clinical Pharmacology, Singapore Health Services, Singapore, Singapore; 2 Division of Medical Oncology, National Cancer Center, Singapore, Singapore; 3 Division of Medical Sciences, National Cancer Center, Singapore, Singapore; 4 Office of Clinical Sciences, Duke-NUS Graduate Medical School, Singapore, Singapore; 5 Institute of Molecular and Cell Biology, A*STAR, Singapore, Singapore; Catalan Institute of Oncology, SPAIN

## Abstract

**Purpose:**

This study aimed to evaluate the safety, tolerability and pharmacokinetics of the combination of oral vinorelbine with erlotinib using the conventional (CSV) and metronomic (MSV) dosing schedules in patients with advanced non-small cell lung cancer (NSCLC).

**Methods:**

This was an open-label, multiple dose-escalation phase I study. An alternating 3+3 phase I design was employed to allow each schedule to enroll three patients sequentially at each dose level. Thirty patients with Stage IIIB/IV NSCLC were treated with escalating doses of oral vinorelbine starting at 40 mg/m^2^ on day 1 and 8 in the CSV group (N = 16) and at 100 mg/week in the MSV group (N = 14). Erlotinib was administered orally daily.

**Results:**

The maximum tolerated dose was vinorelbine 80 mg/m^2^ with erlotinib 100 mg in the CSV group and vinorelbine 120 mg/week with erlotinib 100 mg in the MSV group. Grade 3/4 toxicities included neutropenia (N = 2; 13%) and hyponatremia (N = 1; 6%) in the CSV group, and neutropenia (N = 5; 36%) in the MSV group. Objective response was achieved in 38% and 29% in the CSV and MSV groups respectively. Vinorelbine co-administration did not significantly affect the pharmacokinetics of erlotinib and OSI-420 after initial dose. However, at steady-state, significantly higher C_max_, higher C_min_ and lower CL/F of erlotinib were observed with increasing dose levels of vinorelbine in the CSV group. Significantly higher steady-state C_min_, C_avg_ and AUC_ss_ of erlotinib were observed with increasing dose levels of vinorelbine in the MSV group.

**Conclusions:**

Combination of oral vinorelbine with erlotinib is feasible and tolerable in both the CSV and MSV groups.

**Trial Registration:**

ClinicalTrials.gov NCT00702182

## Introduction

Non-small cell lung cancer (NSCLC) is one of the most common causes of cancer mortality worldwide. Most patients are diagnosed at advanced stages, for which the median survival with best supportive care is approximately 3–6 months [1]. The first-line systemic treatment for patients with advanced NSCLC consists of platinum-based doublet chemotherapy, the survival benefit of which has reached a plateau at approximately 10 months [2], [3] and with a response rate of only about 10% in the salvage settings [4], [5].

Vinorelbine is a semi-synthetic vinca alkaloid which exerts its anti-cancer effects by inhibiting the assembly of microtubules during metaphase in dividing cells. At present, vinorelbine is approved either as a single agent or in combination with cisplatin for the treatment of unresectable, advanced NSCLC. The availability of oral vinorelbine has improved the convenience of its administration. Vinorelbine 60 mg/m^2^ and 80 mg/m^2^ administered orally have been demonstrated to yield equivalent drug exposure to 25 mg/m^2^ and 30 mg/m^2^ of intravenous vinorelbine respectively [6]. However, vinorelbine alone or in combination with cisplatin only modestly improves survival, and more effective treatment approaches are clearly needed for the treatment of advanced NSCLC [7], [8].

The use of epidermal growth factor receptor (EGFR) tyrosine kinase inhibitor (TKI), such as erlotinib and gefitinib, in the treatment of advanced NSCLC has been shown to markedly improve survival, particularly in patients with *EGFR*-activating mutations which are more common in patients of Asian ethnicity [9]. Additive or supra-additive effects have been demonstrated in head and neck squamous cell carcinoma (HNSCC) cell lines concurrently treated with vinorelbine and EGFR TKI [10]. The use of vinorelbine in combination with EGFR TKI, specifically gefitinib [11], [12] have been reported but these combinations were limited by significant myelosuppression. Optimal scheduling of chemotherapy regimens have been shown to be highly critical in maximizing their clinical efficacy and minimizing toxicity.

Metronomic therapy is a novel dosing strategy involving frequent administration of low doses of chemotherapy over prolonged periods of time, and has been demonstrated to affect cycling endothelial cells and inhibit tumour angiogenesis as well as induce apoptosis [13], even in highly resistant tumours [14]. Low-dose metronomic scheduling of chemotherapy agents has also been shown to improve toxicity profiles compared to conventional dosing regimens due to the lower doses administered [15].

Vinorelbine treatment by label is typically given on days 1 and 8 of a 21-day cycle. Continuous administration of metronomic oral vinorelbine in advanced solid tumours, administered thrice per week, has been shown to be feasible and well tolerated up to doses of 150 mg per week [16–19]. However, while several phase I trials have recently defined the maximum tolerated dose (MTD) and safety of metronomic vinorelbine as a single agent or in combination with other chemotherapy agents in patients with advanced cancers [16–19], metronomic vinorelbine in combination with erlotinib has not been explored.

Hence, the primary objective of this study was to assess the safety and tolerability of combining oral vinorelbine and erlotinib on two different schedules: conventional schedule of oral vinorelbine (CSV) given on days 1 and 8 every 21 days plus daily erlotinib and metronomic schedule of oral vinorelbine (MSV) administered thrice weekly plus daily erlotinib. Objective response rates of combining oral vinorelbine and erlotinib administered using the two schedules were also evaluated. The secondary objective was to elucidate the pharmacokinetic interactions between these two drugs under both treatment schedules.

## Materials and Methods

The protocol for this trial as well as supporting TREND checklist are available as supporting information (S1 Protocol and S1 TREND Checklist).

### Eligibility Criteria

Patients aged more than 21 years with histologically or cytologically proven stage IIIB/IV unresectable or metastatic NSCLC who had received at least one prior line of chemotherapy were eligible. Other key inclusion criteria included Eastern Cooperative Oncology Group (ECOG) performance status < 2, life expectancy of at least 3 months and normal organ and marrow function. Prior chemotherapy (≥ 4 weeks before, ≥ 6 weeks if regimen included BCNU or mitomycin C) or radiation therapy (≥ 4 weeks before) were acceptable. Patients were excluded if they had previously received vinorelbine or oral EGFR TKI, were receiving any other investigational agents, had progressive brain metastases or significant malabsorption syndrome affecting gastro-intestinal tract function, uncontrolled co-morbidities including sepsis, arrhythmias, serious non-healing wound, ulcer, bone fracture; history of organ allograft, bleeding diasthesis or coagulopathy and pregnant or breast-feeding. All patients on concomitant treatment with drugs known to induce or inhibit cytochrome p450 (CYP) 3A4, CYP1A1, CYP1A2 were also excluded. Detailed history, including demographics, were collected at baseline. All individual participants provided written informed consent to participate in the study. The study was approved by the ethics review committee of the National Cancer Centre Singapore (IRB Ref: 2007/430/B). ClinicalTrials.gov Identifier: NCT00702182.

### Study Design

**Fig 1:**
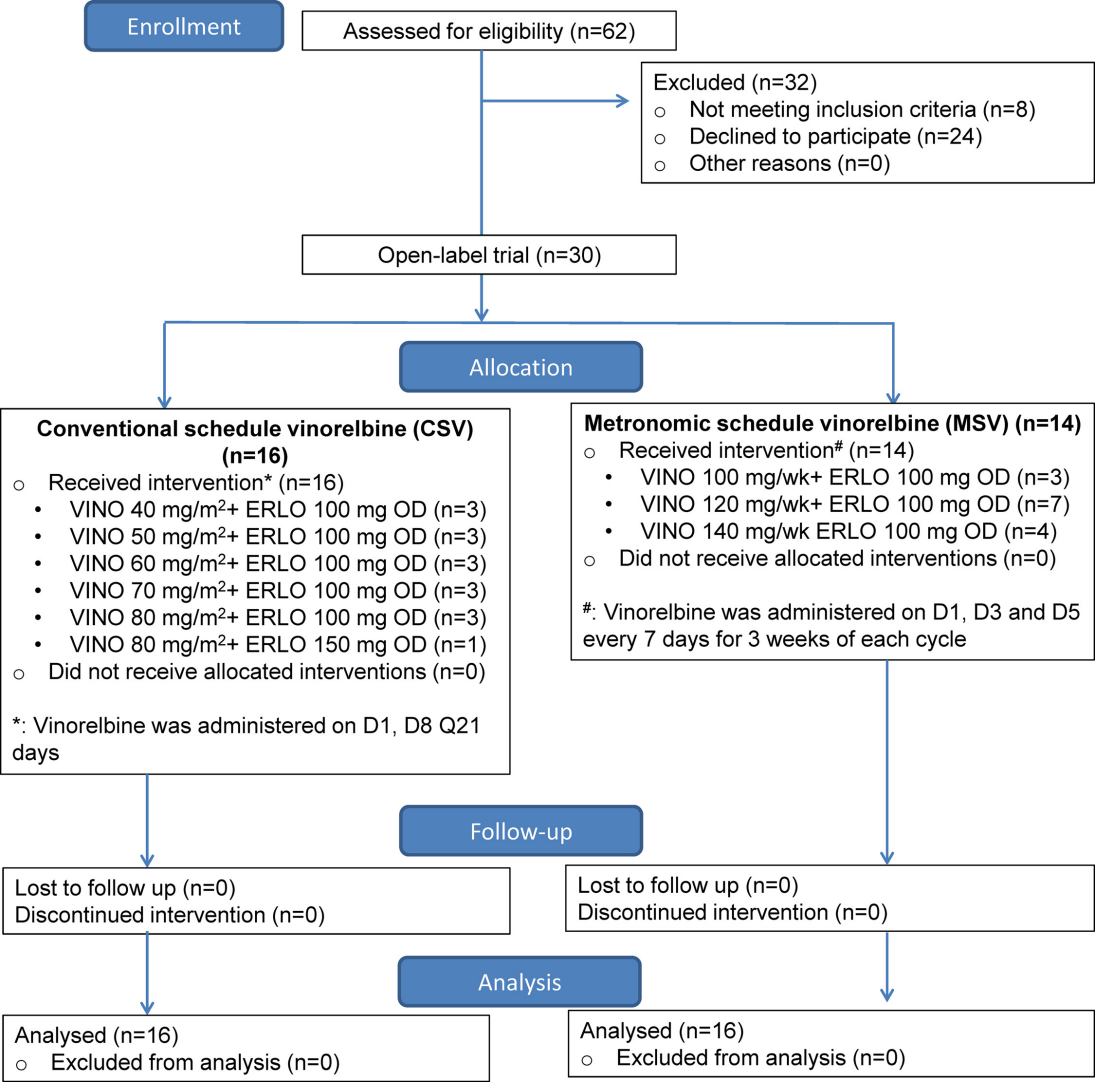
CONSORT Flow diagram.

This was an open-label, single-centre, multiple dose-escalation phase I study conducted at the National Cancer Centre, Singapore, as depicted in the CONSORT flow diagram (Fig 1). Oral vinorelbine was started according to 2 different schedules: conventional schedule vinorelbine (CSV) and metronomic schedule vinorelbine (MSV) (Table 1) two days before starting erlotinib. The standard “3+3” rule was employed for dose escalation. Three patients were treated per cohort. An alternating phase I design was employed, with each schedule accruing sequentially at each dose level. For cycle 1, erlotinib was started 2 days after vinorelbine at 100 mg daily. Escalation of erlotinib to 150 mg daily was permitted if level 5 oral vinorelbine was reached without any dose limiting toxicity (DLT) being observed with erlotinib 100 mg daily in each schedule (Table 1). This design allowed for expedited accrual without compromising safety. In the CSV arm, vinorelbine was administered on Day 1 and Day 8 of each cycle. In the MSV arm, vinorelbine was administered on Day 1, 3, 5 every week of each cycle (Table 1). Each cycle lasted 21 days with a window period of 7 days.

**Table 1:** Dose Escalation Schedule for the CSV and MSV arms.

Erlotinib (mg)(started 2 days after vinorelbine in cycle 1)	CSV	MSV	Dose Level
	Vinorelbine (mg/m^2^) D1, D8 Q21 days	Vinorelbine (mg/week) D1, D3, D5 every 7 days Q21 days	
100	40	100	1
100	50	120	2
100	60	140	3
100	70	160	4
100	80	180	5
Escalate erlotinib to 150 mg if MTD is not reached at dose level 5 of vinorelbine.				
150	80	180	6

Abbreviations: CSV, conventional schedule vinorelbine; MSV, metronomic schedule vinorelbine

Haematological DLT was defined as grade 4 neutropenia lasting more than 7 days duration, neutropenic fever, grade 4 anemia or grade 3–4 thrombocytopenia occurring during the first cycle of treatment. Non-haematological DLT was defined as any grade 3 or 4 non-haematological toxicity occurring during the first cycle of treatment. Any toxicities causing a total of 14 days delay of the next dose were also considered DLTs. No intra-patient dose escalation was allowed. The maximum tolerated dose (MTD) was defined as the dose level at which no more than one patient experienced DLT with at least six patients treated at this dose level.

### Disease Evaluation and Response Assessment

Tumour response measurements were determined by computed tomography (CT) assessment determined at baseline and every 2 cycles. Although not a primary endpoint of this study, patients with measurable disease were assessed using the Response Evaluation Criteria in Solid Tumors (RECIST) criteria. The best overall response was defined as the best response recorded from the start of treatment until disease progression/recurrence.

### Safety Assessment

Toxicities were graded according to the National Cancer Institute Common Terminology Criteria of Adverse Events version 3.0. Toxicities were considered to be related to the study drugs unless they were attributable to either underlying tumour progression, concurrent medical condition, or concurrent medications. Assessment of toxicity was performed at baseline, every 2 weeks during treatment and for 30 days after the last dose of the study drugs, after which only serious adverse events deemed causally related to the study drugs were reported. Any patient who experienced DLT was allowed to continue treatment with a one level dose reduction if toxicity resolved within 14 days. If this patient experienced a DLT at the lowered dose level, study treatment was stopped for that patient. In addition, if the occurrence of a clinically significant toxicity was deemed to be related to either vinorelbine or erlotinib, that study drug could be discontinued in view of patient safety and the patient was allowed to remain in the trial on single agent therapy.

### Pharmacokinetics Analysis

Blood samples (3 mL) were collected at 0 (predose), 0.5, 1, 2, 4, 6, 8, 24 and 48 hr after the initial vinorelbine dose on Day 1. Erlotinib was only started on Day 3 to allow PK profiling of vinorelbine alone. Blood samples were also collected for the PK analysis of erlotinib on Day 3 at 0 (predose), 1, 2, 4, 6, 8, and 24 hr. Steady-state blood samples were collected for both drugs and erlotinib metabolite, OSI-420 on Day 10 at the following time points: 0 (predose), 0.5, 1, 2, 4, 6, 8, and 24 hr. Plasma was immediately harvested from the blood and stored at -80°C until bioanalysis.

The plasma concentrations of vinorelbine were quantified using a modified sensitive liquid chromatography tandem mass spectrometry method previously reported [20]. Briefly, the method involved deproteinization of plasma samples with a mixture of ethanol and acetonitrile, followed by a liquid chromatography coupled through an electrospray interface to a tandem mass spectrometry in positive mode detection. The lower limit of quantitation was 250 pg/mL for vinorelbine. The calibration curve was linear over the concentration range of 0.5–200 ng/mL for vinorelbine. The within-day and between-day coefficients of variation were less than 15% for vinorelbine.

Plasma concentrations of erlotinib and its main metabolite, desmethyl erlotinib (OSI-420), were also quantified using a liquid chromatography tandem mass spectrometry method. Briefly, the method involved liquid-liquid extraction of 50 μL of plasma samples with a mixture of ethyl acetate and n-hexane (8/2, v/v) followed by a liquid chromatography coupled through an electrospray interface to a tandem mass spectrometry in positive mode detection. Chromatographic separation was accomplished using a C18 column (100 mm x 4.6 mm I.D., 5 μm Thermo Hypurity C18, Thermo scientific, USA) with a mobile phase consisting of 2 mM ammonium acetate: methanol (20:80, v/v). The lower limit of quantitation for erlotinib and OSI-420 were 10.4 and 2.3 ng/ml, respectively. The calibration curve was linear over a concentration range of 10.4–2510.8 ng/mL for erlotinib and 2.3–562.5 ng/mL for desmethyl erlotinib. The within-day and between-day coefficients of variation were both less than 15%. PK parameters were calculated by non-compartmental analysis with linear trapezoidal method using WinNonlin Version 6.3 (Pharsight Corp, Tripos, L.P).

### Statistical Analysis

One-way ANOVA was applied to compare the PK parameters between different dose groups of vinorelbine with Graphpad Prism 6 (GraphPad Software, Inc., La Jolla, CA, USA). The overall survival and progression free survival in the CSV and MSV groups were evaluated using Graphpad Prism 6. *P* value of less than 0.05 was considered statistically significant.

## Results

### Patient Characteristics

Thirty patients were recruited between April 2008 and March 2011, out of which 16 were recruited into the CSV arm and 14 were recruited into the MSV arm. Patient characteristics are listed in Table 2. Patient demographics in terms of age, gender and ethnicity distribution between the two groups were comparable. In the CSV arm (N = 16), only 1 patient had ECOG 0 and the remaining 15 patients had ECOG 1. The majority of patients (N = 11; 69%) had adenocarcinoma, with the remaining having squamous cell carcinoma histology (N = 3; 19%) or other histological subtypes of NSCLC (N = 2; 12%). Half of the patients (N = 8; 50%) had received prior radiotherapy. All patients had at least one prior line of chemotherapy. Most patients (N = 10; 63%) were never-smokers.

**Table 2:** Patient demographics and baseline characteristics.

Characteristics	CSV (n = 16)	MSV (n = 14)
**Gender, n (%)**		
Male	9 (56)	6 (43)
Female	7 (44)	8(57)
**Median age (years, range)**	59 (39–73)	55 (33–72)
**Ethnicity, n (%)**		
Chinese	14 (88)	12 (86)
Malay	1 (6)	1 (7)
Indian	0 (0)	1 (7)
Others	1 (6)	0 (0)
**ECOG performance status, n (%)**		
0	1 (6)	2 (14)
1	15 (94)	12 (86)
**Histology, n (%)**		
Adenocarcinoma	11 (69)	8 (57)
Squamous	3 (19)	3 (21)
Others	2 (12)	3 (21)
**Prior radiotherapy, n (%)**		
Yes	8 (50)	6 (43)
No	8 (50)	8 (57)
**Prior chemotherapy, n (%)**		
Yes	16 (100)	14 (100)
No	0 (0)	0 (0)
**No. of prior chemotherapy regimens, n (%)**		
1	8 (50)	4 (29)
2	6 (38)	8 (57)
3	2 (13)	2 (15)
**Smoking history, n (%)**		
Current smoker	2 (13)	1 (7)
Never smoker	10 (63)	10 (71)
Ex-smoker	4 (25)	3 (21)

Abbreviations: ECOG, Eastern Cooperative Oncology Group

In the MSV arm, 2 of 14 patients (N = 2; 14%) had ECOG 0 and the remaining 12 patients had ECOG 1. Most patients (N = 8; 57%) had adenocarcinoma, while 21% (N = 3) each had squamous cell histology or other NSCLC. Only 43% (N = 6) of patients had received prior radiotherapy but similar to the CSV arm, all patients had received at least one prior line of chemotherapy, with the majority (N = 8; 57%) having received 2 or more previous lines. Similar to the CSV arm, most patients (N = 10; 71%) in the MSV arm were never-smokers.

### Dose Escalation and Toxicities

In the CSV arm, we encountered no DLTs in patients treated at dose levels 1 to 5. One patient at dose level 4 was admitted with grade 3 neutropenia, shortness of breath and vomiting, but this occurred after 4 cycles and was not categorized as a DLT. One patient was subsequently recruited and treated with vinorelbine 80 mg/m^2^ on days 1 and 8 every 21 days plus daily erlotinib 150 mg. This patient was identified to have grade 4 hyponatremia on day 8 of cycle 1. Treatment was interrupted and the patient subsequently had left-sided lung collapse unrelated to the study drugs a month after resolution of hyponatremia and passed on from disease-related complications beyond cycle 1. No further patients were recruited at this dose level because the introduction of routine *EGFR* mutation profiling and first-line treatment of patients with *EGFR* mutated disease made further recruitment untenable.

In the MSV arm, three patients were treated with no DLTs at dose level 1. One patient at dose level 1 had grade 3 neutropenia which did not occur in cycle 1 of treatment and hence was not categorized as a DLT. In dose level 2, four patients were initially treated with no DLTs, and accrual proceeded to dose level 3. Four patients were treated at dose level 3. The first patient had grade 4 neutropenia with sepsis but this occurred after cycle 2 and was not categorized as DLT. The second patient was admitted for grade 4 neutropenia and developed febrile neutropenic episode requiring granulocyte colony-stimulating factor, which was considered DLT. The third patient recruited had grade 3 neutropenia with fever on day 15 in cycle 2 of treatment and was thus not considered DLT. The next patient enrolled was admitted for grade 4 febrile neutropenia on day 15 of cycle 1. Hence, two of the four patients enrolled at dose level 3 had DLTs. Three additional patients were enrolled at dose level 2. No DLTs occurred and thus the MTD of vinorelbine in combination with erlotinib in the MSV arm was established as vinorelbine 120 mg per week and erlotinib 100 mg daily on a 21-day cycle.

Table 3 lists all the adverse events that were at least possibly related to either vinorelbine or erlotinib throughout all treatment cycles. Toxicities were generally mild and the most commonly observed AEs were dermatological toxicities [dry skin, 100% (CSV), 93% (MSV); rash, 94% (CSV), 86% (MSV); pruritus, 88% (CSV), 86% (MSV)]. Acneiform rash, which is a common adverse effect of erlotinib, was common but none were above grade 2. Gastrointestinal toxicities were also common, with grade 1 to 2 diarrhoea occurring in 63% and 79% of patients in the CSV and MSV arms, respectively. Hematologic toxicity consisted of only neutropenia in the CSV arm, with two patients (13%) developing grade 3 neutropenia.

**Table 3:** Adverse events in all cycles of treatment for all patients receiving at least one cycle of treatment in the CSV and MSV groups.

Adverse Event	CSV		MSV	
	All grades, n (%)	Grades 3/4, n (%)	All grades, n (%)	Grades 3/4, n (%)
**Gastrointestinal**				
Diarrhea	10 (63)	0 (0)	11 (79)	1 (7)
Nausea	3 (19)	0 (0)	6 (43)	0 (0)
Vomiting	4 (25)	0 (0)	1 (7)	0 (0)
Constipation	4 (25)	0 (0)	1 (7)	0 (0)
Dysphagia/ heartburn	2 (13)	0 (0)	0 (0)	0 (0)
Stomatitis	6 (38)	0 (0)	3 (21)	0 (0)
Anorexia/loss of appetite	0 (0)	0 (0)	3 (21)	0 (0)
**Dermatological**				
Rash	15 (94)	0 (0)	12 (86)	0 (0)
Pruritus	14 (88)	0 (0)	12 (86)	0 (0)
Dry skin	16 (100)	0 (0)	13 (93)	0 (0)
Alopecia	0 (0)	0 (0)	1 (7)	0 (0)
**Metabolic**				
Hyponatremia	1 (6)	1 (6)	0 (0)	0 (0)
**Hematologic/Infection**				
Anemia	0 (0)	0 (0)	2 (14)	0 (0)
Non-febrile neutropenia	2 (13)	2 (13)	1 (7)	1 (7)
Infection with normal ANC	0 (0)	0 (0)	1 (7)	0 (0)
Neutropenic fever	0 (0)	0 (0)	4 (29)	4 (29)
**Pain**				
Nose	1 (6)	0 (0)	0 (0)	0 (0)
Face	1 (6)	0 (0)	0 (0)	0 (0)
Joint	1 (6)	0 (0)	0 (0)	0 (0)
Muscle	0 (0)	0 (0)	1 (7)	0 (0)
Nail	0 (0)	0 (0)	1 (7)	0 (0)
**Others**				
Dry eyes	0 (0)	0 (0)	3 (21)	0 (0)
Keratitis	0 (0)	0 (0)	2 (14)	0 (0)
Fatigue	5 (31)	0 (0)	6 (43)	1 (7)
Giddiness	1 (6)	0 (0)	0 (0)	0 (0)
Neuropathy	4 (25)	0 (0)	2 (14)	0 (0)
Paronychia	2 (13)	0 (0)	5 (36)	0 (0)

Note: Each adverse event is reported once, at the maximum grade, for each patient. S1 Table and S2 Table list the toxicities by dose levels in both treatment groups.

In contrast, grade 3 to 4 neutropenia occurred more frequently in the MSV arm (36%). Of the five patients with grade 3 to 4 neutropenia, four patients (29%) had febrile neutropenia. Notably, all four of these patients were receiving the maximum administered vinorelbine dose of 140 mg per week. Other hematologic toxicities in the MSV arm consisted of grade 1 to 2 anemia, which occurred in two patients (14%), and grade 2 infection with normal absolute neutrophil count, which occurred in one patient (7%). Neuropathy and paronychia were common in both the CSV and MSV arms, but all episodes were grade 1 to 2. Fatigue was also common in both arms, but only one patient (7%) in the MSV arm had grade 3 fatigue. There were no treatment-related deaths reported in either arm. S1 Table and S2 Table list all the toxicities by dose levels in both treatment groups.

### Efficacy

While this study was not powered for evaluation of anti-cancer efficacy, all 30 patients had their best objective response rate evaluated at the end of treatment (Table 4). A total of 6 patients (37.5%) in the CSV arm had partial response. Eight patients (50%) had stable disease and 2 patients (12.5%) progressed. In the MSV arm, 4 patients (28.6%) had partial response and 5 patients (35.7%) had stable disease while another 5 patients (35.7%) progressed.

**Table 4:** Best response rate by dose level.

Dose level	Vinorelbine dose	Erlotinib dose (mg/day)	No. of enrolled patients	Best overall response (n)			Response rate
				PR	SD	PD	
**CSV Vinorelbine (administered on D1, D8 Q21)**							
1	40 mg/m^2^	100	3	1	2	0	33.3%
2	50 mg/m^2^	100	3	0	1	2	0.0%
3	60 mg/m^2^	100	3	1	2	0	33.3%
4	70 mg/m^2^	100	3	2	1	0	66.6%
5	80 mg/m^2^	100	3	2	1	0	66.6%
6	80 mg/m^2^	150	1	0	1	0	0.0%
Total			16	6	8	2	
**Total rate**				**37.5%**	**50%**	**12.5%**	
**MSV Vinorelbine (administered D1, D3, D5 every week Q21)**							
1	100	100	3	1	1	1	33.3%
2	120	100	7	2	1	4	28.6%
3	140	100	4	1	3	0	25.0%
Total			14	4	5	5	
**Total rate**				**28.6%**	**35.7%**	**35.7%**	

### Pharmacokinetics

**Fig 2:**
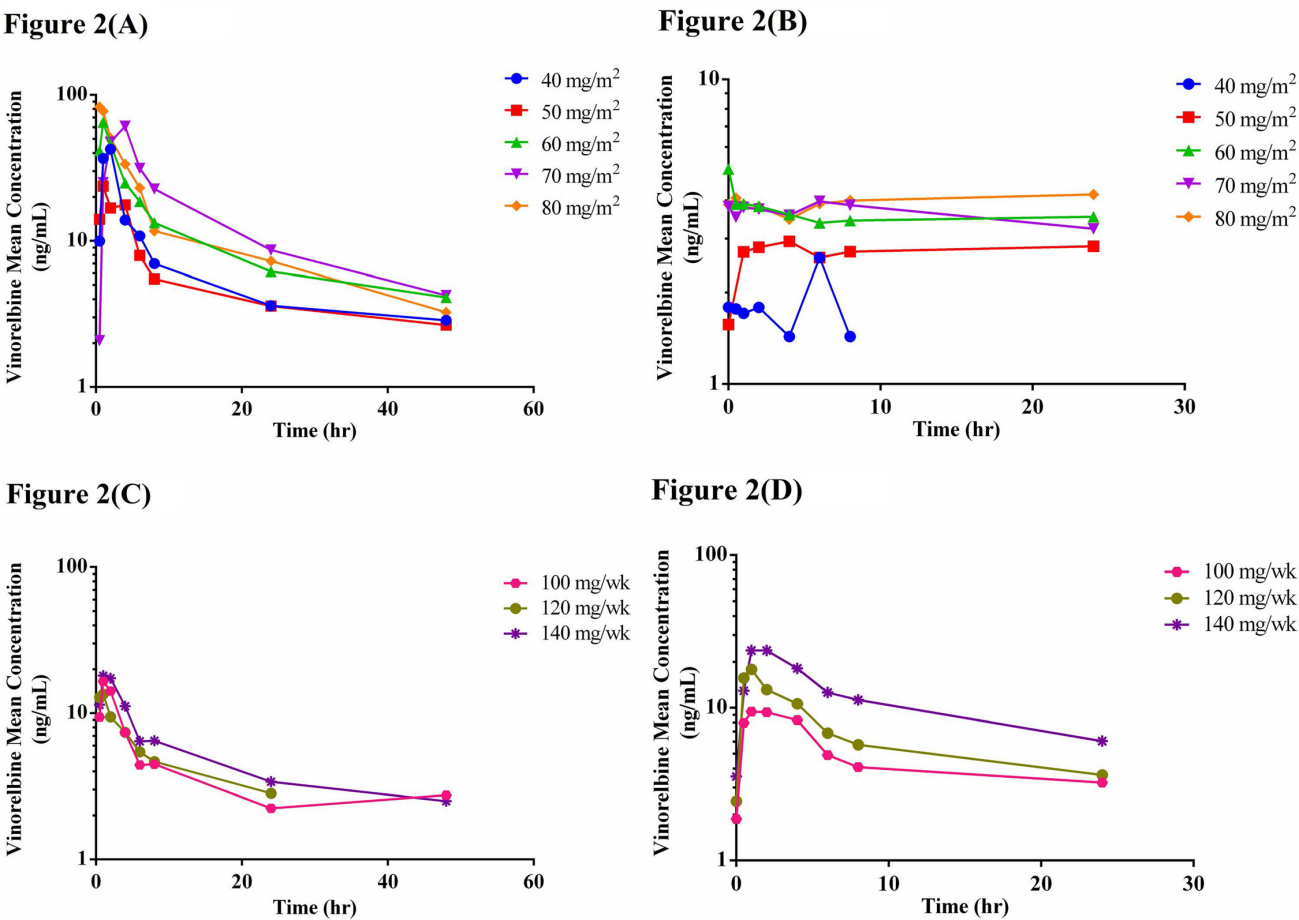
Mean plasma concentration-time curves of vinorelbine (A) on Day 1 after initial oral dose with CSV schedule, (B) on Day 10 at steady state after oral dose with CSV schedule, (C) on Day 1 after initial oral dose with MSV schedule, and (D) on Day 10 at the steady state after oral dose with MSV schedule.

**Fig 3:**
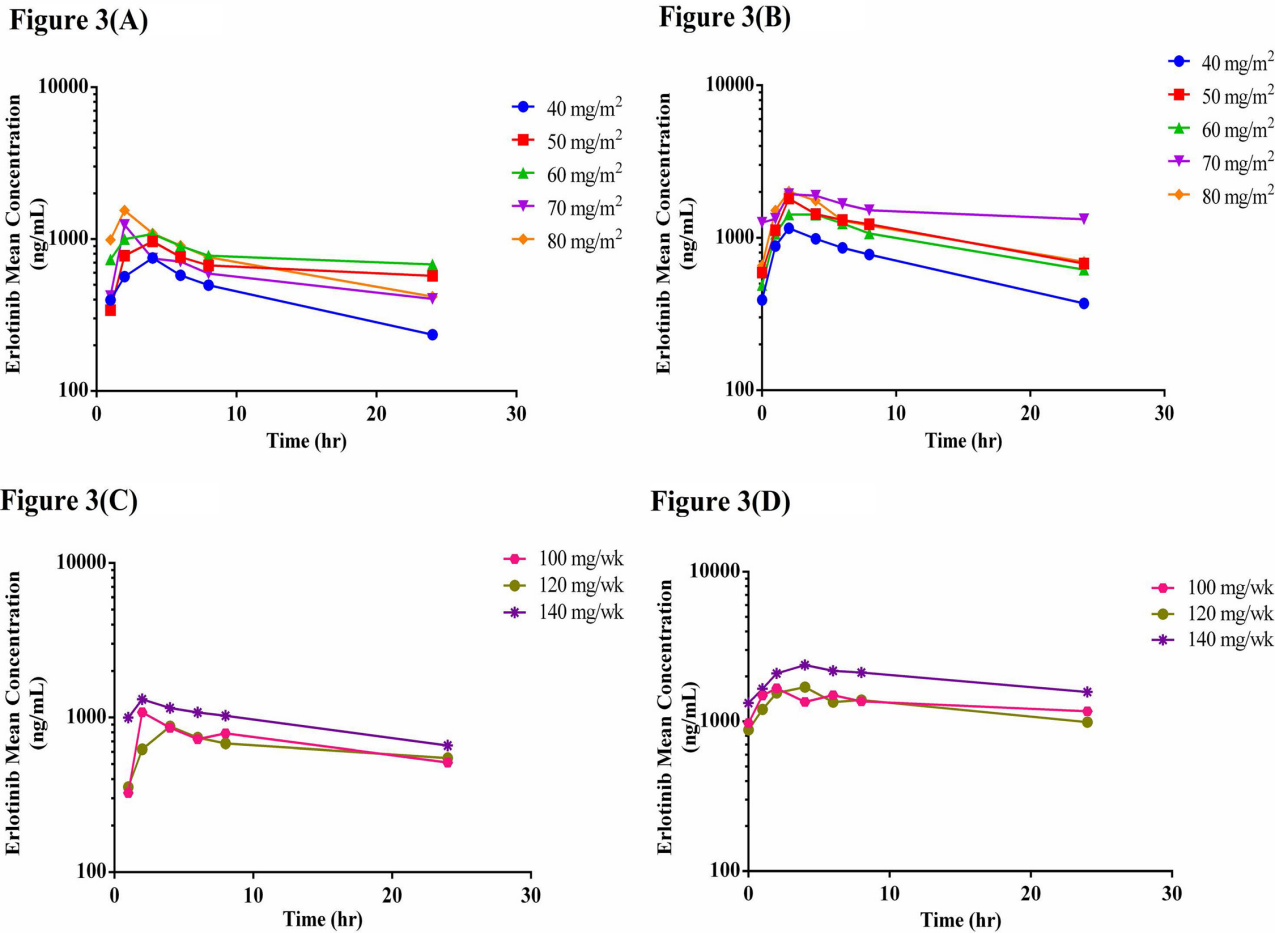
Mean plasma concentration-time curves of erlotinib (A) on Day 3 after initial oral dose with CSV dosing regimen of vinorelbine, (B) on Day 10 at the steady state after oral dose with CSV dosing regimen of vinorelbine, (C) on Day 3 after initial oral dose with MSV dosing regimen of vinorelbine and (D) on Day 10 at the steady state after oral dose with MSV dosing regimen of vinorelbine.

**Fig 4:**
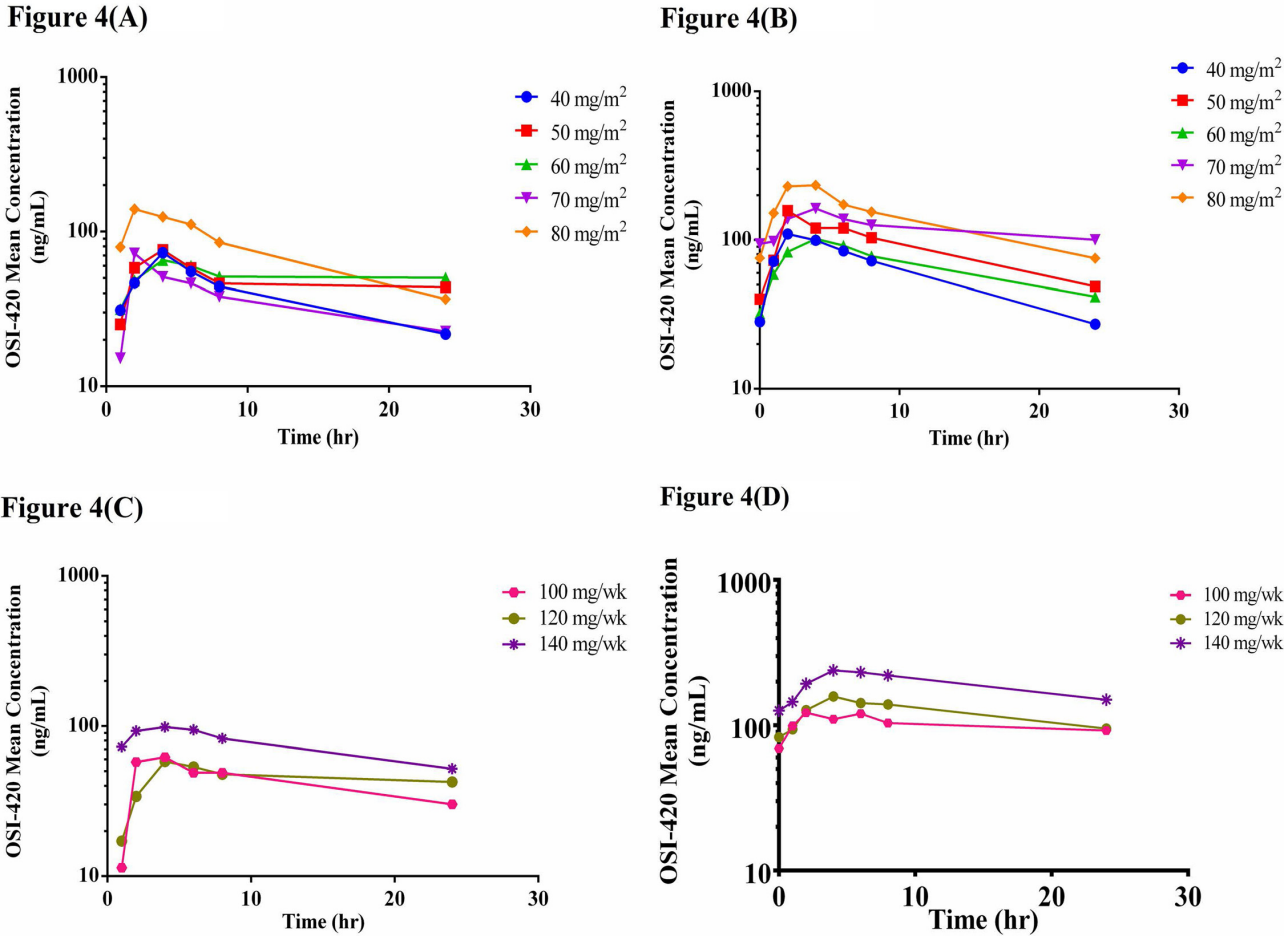
Mean plasma concentration-time curves of OSI-420 (A) on Day 3 after initial oral dose with CSV dosing regimen of vinorelbine, (B) on Day 10 at the steady state after oral dose with CSV dosing regimen of vinorelbine, (C) on Day 3 after initial oral dose with MSV dosing regimen of vinorelbine and (D) on Day 10 at the steady state after oral dose with MSV dosing regimen of vinorelbine.

The pharmacokinetics of vinorelbine, erlotinib, and OSI-420 were investigated after the initial doses as well as at steady state in all patients recruited to the CSV (N = 16) and MSV (N = 14) groups (Figs 2–4). The PK parameters after the first dose of vinorelbine as well as at steady state in the CSV and MSV groups were calculated. The PK parameters were also calculated for erlotinib and its metabolite OSI-420 after the initial dose of erlotinib on Day 3 and at its steady state in both the CSV and MSV groups.

Following the first dose of erlotinib, exposure to erlotinib in terms of C_max_ and AUC_tau_ was not significantly different between patients receiving different dose levels of vinorelbine in neither the CSV nor MSV groups (*P* > 0.05). Interestingly, however, at steady state, significant differences were observed in the C_max_ (*P* = 0.03), C_min_ (*P* = 0.01), CL/F (*P* = 0.02), and accumulation factor (*P* = 0.02) of erlotinib between the different dose levels of vinorelbine in the CSV group. Specifically, we observed trends of higher C_max_, higher C_min_ and lower CL/F of erlotinib with increasing dose levels of vinorelbine. Similarly, significant differences were observed on the PK parameters of erlotinib (C_min_, C_avg_, and AUC_ss_ with *P*-values of 0.05, 0.04, and 0.04, respectively) between the different dose levels of vinorelbine in the MSV group. Similarly for the MSV group, trends towards higher C_min_, C_avg_ and AUC_ss_ were observed with increasing dose levels of vinorelbine.

The PK parameters of OSI-420, C_max_ and AUC_tau_, after the first dose of erlotinib were similar at all vinorelbine dose levels in both the CSV and MSV groups (*P* > 0.05). At the steady state, only the C_min_ (*P* = 0.03) and accumulation factor (*P* = 0.01) of OSI-420 showed statistically significant difference between different doses of vinorelbine with the CSV dosing regimen but not with the MSV regimen.

Post hoc tests following one-way ANOVA was used to conduct multiple comparisons between each dose group and every other dose group. At the steady state of erlotinib, C_max_, C_min_, C_avg_, AUC_ss_, accumulation factor, and CL/F for erlotinib were significantly different in patients receiving 40 mg/m^2^ and 70 mg/m^2^ in the CSV group. For OSI-420 at the steady state, accumulation factor is significantly different between 40 and 70 mg/m^2^ CSV groups. The C_max_ for erlotinib at the steady state was significantly different in patients receiving 40 and 80 mg/m^2^ in the CSV group. The C_min_, C_avg_, and AUC_ss_ of erlotinib at the steady state were significantly different between patients receiving 120 mg/week and 140 mg/week in the MSV group.

## Discussion

This study has demonstrated that the combination of erlotinib and oral vinorelbine administered in both the CSV and MSV schedules is feasible and tolerable in patients with advanced NSCLC who had previously failed standard chemotherapy. The MTDs were vinorelbine 80 mg/m^2^ on Day 1 and Day 8 with erlotinib 100 mg/day every 21 days in the CSV group and vinorelbine 40 mg on Days 1, 3 and 5 weekly (120 mg/week) with erlotinib 100 mg/day every 21 days in the MSV group. The safety profile of the combination was consistent with each of the individual drug, with no unexpected toxicities observed during the study.

The combination of erlotinib and vinorelbine administered intravenously in the conventional manner has previously been investigated in another phase I study by Davies et al. [21]. They reported an MTD of vinorelbine 25 mg/m^2^ administered intravenously on Days 1 and 8 with erlotinib 100 mg/day every 21 days [21]. In contrast to the low incidence of haematological toxicities observed in the CSV group in our study, the investigators noted a high incidence of grade 3/4 neutropenia (50%) and febrile neutropenia (25%), which occurred in patients receiving intravenous vinorelbine 25 mg/m^2^ with erlotinib 150 mg. The reason for such discrepancy is unclear, although it may be due in part to the inclusion of patients with poorer performance status in the study by Davies et al., with 25% of recruited patients having performance status of <80.

In addition, both vinorelbine and erlotinib are substrates of CYP3A4. Although erlotinib does not commonly cause haematological toxicities, it is possible that erlotinib at a higher dose of 150 mg may elevate the systemic exposure of vinorelbine as compared to erlotinib at lower dose of 100 mg, which may explain the high incidence of haematological toxicities in the study by Davies et al. [21]. Unfortunately, since only 1 patient in the CSV group receiving 80 mg/m^2^ vinorelbine and 150 mg erlotinib was recruited and none of the patients in the MSV group received 150 mg erlotinib, this could not be supported by the pharmacokinetics findings of this study.

Several trials have investigated the safety and tolerability of metronomic vinorelbine either as a monotherapy or in combination with other chemotherapy agents. However, to the best of our knowledge, this is the first study investigating the combination of metronomic oral vinorelbine with erlotinib in NSCLC patients. Optimal scheduling and dosing of chemotherapy have been shown to be extremely important both in terms of safety and efficacy. Our intention was therefore to explore the role of the combination of oral vinorelbine administered in a metronomic manner with erlotinib in patients with advanced NSCLC. We found that metronomic oral vinorelbine up to doses of 40 mg thrice weekly (120 mg/week) with erlotinib 100 mg/day was very well tolerated, with no DLTs reported.

However, all the four patients who received higher vinorelbine dose of 140 mg/week with erlotinib 100 mg/day experienced grade 3/4 neutropenic fever, two of which were considered DLTs. These findings were in contrast with those noted by Pallis et al. and Briasoulis et al., which reported that the administration of oral vinorelbine doses up to 60 mg every other day and 50 mg thrice weekly in each cycle respectively was characterized by acceptable toxicities [17], [22]. Moreover, Pallis et al. conducted the study in patients receiving oral vinorelbine in combination with cisplatin, while Briasoulis et al. conducted a study in which vinorelbine was administered as a monotherapy [17], [22]. One possible explanation for the observed haematological toxicity at this dose level (vinorelbine 140 mg/week plus erlotinib 100 mg/day) in our study but not others could be due to the altered pharmacokinetics of vinorelbine at steady state when administered together with erlotinib, both of which are substrates of CYP3A4. Indeed, the exposure to vinorelbine in cycle 1 in patients with grade 3/4 neutropenia was shown to be higher than those who did not experience neutropenia in the MSV group. However, this trend fell short of achieving statistical significance, possibly due to the small sample size in this study.

Although the co-administration of vinorelbine with either the CSV or MSV dosing regimen did not have any significant effects on PK parameters of erlotinib and OSI-420 after initial dose, the co-administration of vinorelbine in the CSV group affected PK parameters of erlotinib as well as the metabolite, OSI-420 at steady state. Similarly, co-administration of vinorelbine with MSV dosing regimen affected PK parameters of erlotinib at the steady state. Nevertheless, there were no significant differences in exposure to erlotinib in patients who developed diarrhoea, which has been associated with high levels of exposure to erlotinib, than in those who did not develop diarrhoea.

Although not a primary end-point, we also evaluated the efficacy of this drug combination in both the CSV and MSV groups. Interestingly, patients in the CSV group showed better tumour response than those in the MSV group. Although it would be premature to conclude based on the results of this study alone, given the limited data and nature of phase I studies, it is possible that peak plasma concentrations of vinorelbine need to reach certain therapeutic thresholds for its anti-tumour activities to be effective, which could not be achieved with low-dose metronomic dosing. Nonetheless, in line with the encouraging data reported by other investigators on the anti-cancer efficacy of metronomic vinorelbine in several types of cancer [16], [17], [22], [23], the overall response rate in the MSV group in this study was 29%, with median PFS of 7.5 months and median OS of 11.0 months. These findings were comparable to those of Del Conte et al. which evaluated the tolerability and efficacy of metronomic vinorelbine monotherapy in lung cancer patients [24]. The investigators reported a similar objective response rate of 29%, with median PFS and OS of 4.5 and 11.1 months respectively [24].

However, it is not clear if the addition of erlotinib to the treatment regimen provided any additional clinical benefits. Indeed, most clinical studies to date have failed to demonstrate significant survival benefits in combining platinum-based doublet chemotherapy with EGFR TKIs both in the first-line settings and following disease progression on first-line EGFR TKIs [25–28]. Recently, however, some phase II studies have shown potential benefits of EGFR TKI treatment combined with chemotherapy in patients harbouring sensitizing *EGFR* mutations [29], [30]. It is also noteworthy that studies investigating the potential benefits of pharmacodynamics separation using intercalated dosing schedules of EGFR TKIs in combination with platinum-based doublet chemotherapy have recently reported promising results [31]–[33]. This includes the results of the subgroup analysis of the FASTACT-2 study, which showed significantly longer PFS and OS in patients receiving the combination of gemcitabine and carboplatin with intercalated erlotinib compared to patients receiving chemotherapy alone [32].

It is important to note that this trial was conducted in an unselected patient population and that molecular biomarkers, in particular the presence of activating *EGFR* mutations which have been shown to influence treatment outcomes to EGFR TKIs, were not used as selection criteria for recruitment of patients into the trial. Since the conclusion of the trial, multiple randomized trials have confirmed the superiority of EGFR TKIs over standard chemotherapy in NSCLC patients carrying sensitizing *EGFR* mutations [34–36]. Given the high percentage of recruited patients who were never smokers in both the CSV and MSV groups and the high prevalence of *EGFR* mutations reported in Asians and non-smokers, it is likely that most of these patients harboured sensitizing *EGFR* mutations. Nevertheless, future trials must take into account *EGFR* mutation status, which is now routinely assessed. Considering the safety and tolerability profile of the combination of oral vinorelbine and erlotinib shown in this study, further investigations are warranted to explore the clinical application of this combination, particularly in patients harbouring *EGFR* mutations who are not candidates for more aggressive treatment options.

## Conclusion

This study showed that the combination of oral vinorelbine and erlotinib is safe and tolerable in patients with advanced NSCLC at the following MTDs: in the CSV schedule, vinorelbine 80 mg/m^2^ with erlotinib 100 mg; in the MSV schedule, vinorelbine 40 mg thrice weekly (120 mg/week) with erlotinib 100 mg. Further investigations are warranted to fully elucidate the efficacy and safety of this combination in NSCLC patients carrying activating *EGFR* mutations.

## Supporting Information

S1 TREND Checklist TREND statement checklist. (DOC)

S1 Protocol Clinical trial protocol. (PDF)

S1 Table Adverse events in all cycles of treatment for patients in each dose level of the CSV group. (DOCX)

S2 Table Adverse events in all cycles of treatment for patients in each dose level of the MSV group. (DOCX)
