# Supplementary material for: Phase I Study of Oral Vinorelbine in Combination with Erlotinib in Advanced Non-Small Cell Lung Cancer (NSCLC) Using Two Different Schedules
Source: PLoS One. 2016 May 2;11(5):e0154316. doi: 10.1371/journal.pone.0154316 (PMC4852941; doi:10.1371/journal.pone.0154316)
Supplement: S1 Protocol — (PDF) [file pone.0154316.s002.pdf]

**PROTOCOL TITLE:** A Phase 1 Study of Oral Vinorelbine in Combination with Erlotinib in Advanced Non-Small Cell Lung Cancer (NSCLC) using Two Different Schedules

**Protocol Type / Version # / Version Date:** *Version # 6.0 / 09 Apr 2010*

## SCHEMA

Patients must have histologically or cytologically confirmed stage IIIB/IVNSCLC
One or two prior lines of chemotherapy
Age > 21 years.
ECOG performance status <2 (Karnofsky >60%)
Life expectancy of greater than 3 months
Patients must have normal organ and marrow function

↓

PK of Vinorelbine and Erlotinib

| **Dose Escalation Schedule (see protocol for details)** | | | |
|---|---|---|---|
| | **Dose** | | |
| | **Conventional Schedule Vinorelbine (CSV)**<br>*Vinorelbine (mg/m$^2$ on D1,D8 every 21 days)* | **Metronomic Schedule Vinorelbine (MSV)**<br>*Vinorelbine (mg/week)* | **Dose Level** |
| **Erlotinib 100 mg** (start 2 days after vinorelbine for cycle 1) | 30 | 80 | Level -1 |
| | 40 | 100 | Level 1 |
| | 50 | 120 | Level 2 |
| | 60 | 140 | Level 3 |
| | 70 | 160 | Level 4 |
| | 80 | 180 | Level 5 |

↓

Establish MTD with 100 mg Erlotinib

↓

Escalate Erlotinib to 150 mg (if MTD not reached at level 5 of vinorelbine)

| | | | |
|---|---|---|---|
| **Erlotinib 150 mg** (if no DLT at dose level 5 vinorelbine) | DLT not reached at dose level 5 of vinorelbine (80 mg/m2) | DLT not reached at dose level 5 of vinorelbine (180 mg) | |

## TABLE OF CONTENTS


Page

**SCHEMA** ... i

**1. OBJECTIVES** ... 1
- 1.1 **Primary Objectives** ... 1
- 1.2 **Secondary Objectives** ... 1

**2. BACKGROUND** ... 1
- 2.1 **Drugs** ... 1
- 2.2 **NSCLC** ... 20
- 2.3 **Rationale** ... 20

**3. PATIENT SELECTION** ... 21
- 3.1 **Eligibility Criteria** ... 21
- 3.2 **Exclusion Criteria** ... 22
- 3.3 **Inclusion of Women and Minorities** ... 23

**4. REGISTRATION PROCEDURES** ... 23

**5. TREATMENT PLAN** ... 23
- 5.1 **Agent Administration** ... 23
- 5.2 **Definition of Dose-Limiting Toxicity** ... 27
- 5.3 **General Concomitant Medication and Supportive Care Guidelines** ... 27
- 5.4 **Duration of Therapy** ... 27
- 5.5 **Duration of Follow Up** ... 28
- 5.6 **Criteria for Removal from Study** ... 28

**6. DOSING DELAYS/DOSE MODIFICATIONS** ... 28

**7. ADVERSE EVENTS: LIST AND REPORTING REQUIREMENTS** ... 28
- 7.1 **Adverse Event Characteristics** ... 29
- 7.2 **Expedited Adverse Event Reporting** ... 29
- 7.3 **Routine Adverse Event Reporting** ... 30
- 7.4 **Secondary AML/MDS** ... 30

**8. PHARMACEUTICAL INFORMATION** ... 30
- 8.1 **Oral Vinorelbine** ... 30
- 8.2 **Erlotinib** ... 31

**9. CORRELATIVE/SPECIAL STUDIES** ... 31

- 9.1 Rationale for correlative study ... 31

**10. STUDY CALENDAR** ........................................................................ 32

**11. MEASUREMENT OF EFFECT** ........................................................................ 34
11.1 **Antitumor Effect – Solid Tumors** ........................................................................ 34

**12. DATA REPORTING / REGULATORY REQUIREMENTS** ........................................................................ 37
12.1 **Definition of Adverse Events** ........................................................................ 37
12.2 **Reporting of Adverse Events** ........................................................................ 37
12.3 **Definition of Serious Adverse Events** ........................................................................ 38
12.4 **Expedited Reporting Guidelines** ........................................................................ 38

**13. STATISTICAL CONSIDERATIONS** ........................................................................ 38
13.1 **Analysis** ........................................................................ 38
13.2 **Sample Size Consideration** ........................................................................ 38

**REFERENCES** ........................................................................ 39

**APPENDIX A**
Performance Status Criteria ........................................................................ 40

## 1. OBJECTIVES

1.1 Primary Objectives

- To determine the Maximum Tolerated Dose (MTD) of conventional schedule oral vinorelbine (CSV) combined with erlotinib in patients with NSCLC.
- To determine the Maximum Tolerated Dose (MTD) of metronomic schedule of oral vinorelbine (MSV) combined with erlotinib in patients with NSCLC.

1.2 Secondary Objectives

- To assess for pharmacokinetic interaction of erlotinib on navelbine
- To assess the usefulness of circulating endothelial cells (CECs) and circulating endothelial progenitor cells (CEPs) as surrogate biomarkers to determine optimal anti-angiogenic effect of treatment
- To evaluate the clinical benefit rate (CR+PR+SD) and overall survival

## 2. BACKGROUND

### 2.1 <u>Drugs</u>

#### 2.1.1 Oral Vinorelbine

**Description**

NAVELBINE® ORAL

**Vinorelbine 20 mg and 30 mg soft capsules.**

Vinorelbine tartrate is a semi-synthetic vinca alkaloid with antitumor activity. The chemical name is 3',4'-didehydro-4'-deoxy-C'-norvincaleukoblastine [*R-(R*,R*)*-2,3 dihydroxybutanedioate (1:2)(salt)].

Vinorelbine tartrate has the following structure:

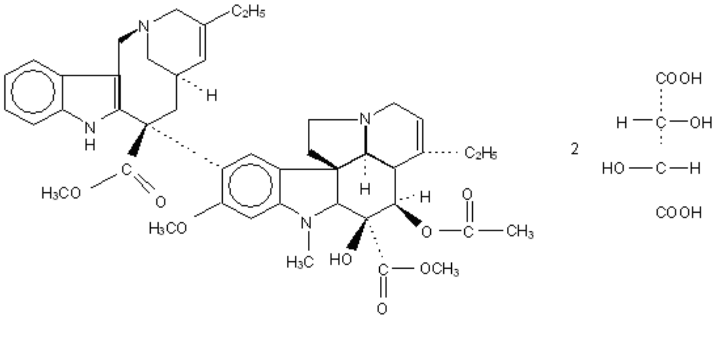


CAS No: 125317-39-7

Vinorelbine tartrate is a white to yellow or light brown amorphous powder with the molecular formula $C_{45}H_{54}N_4O_8.2C_4H_6O_6$ and molecular weight of 1079.12. The aqueous solubility is >

1000 mg/mL in distilled water.
NAVELBINE® soft capsules also contains the following excipients: ethanol, water - purified, glycerol, macrogol 400, gelatin, sorbitol, sorbitan, medium-chain triglycerides, phosphatidyl choline, glycerides, hypromellose, propylene glycol, edible printing ink E120, titanium dixoide, iron oxide yellow CI77492 and / or iron oxide red CI77491.

## Pharmacology

Vinorelbine is an antineoplastic drug. It is a semi-synthetic member of the vinca alkaloid family that interferes with microtubule assembly. The vinca alkaloids are structurally similar compounds comprised of two multiringed units, vindoline and catharanthine. Unlike other vinca alkaloids, the catharanthine unit is the site of structural modification for vinorelbine. The antitumor activity of vinorelbine is thought to be due primarily to inhibition of mitosis at metaphase through its interaction with tubulin. In intact tectal plates from mouse embryos, vinorelbine, vincristine, and vinblastine inhibited mitotic microtubule formation at the same concentration (2µM), including a blockade of cells at metaphase. Vincristine produced depolymerisation of axonal tubules at 5µM, but vinblastine and vinorelbine did not have this effect until concentrations of 30µM and 40µM respectively. These data suggest relative selectivity of vinorelbine for mitotic microtubules.

## Pharmacokinetics

Following oral administration, NAVELBINE® is promptly absorbed and the $T_{max}$ is reached within 1.5 to 3 hours with a blood concentration peak ($C_{max}$) of approximately 130 ng/mL after dosing at 80 mg/m2. The absolute bioavailability is about 40% and simultaneous intake of a low fat standard meal does not modify the area-under the concentration-time curve (AUC). The effect of a high fat meal on absorption has not been studied.

NAVELBINE® Oral 60 and 80 mg/$m^2$ leads to a comparable AUC to that obtained from 25 and 30 mg/$m^2$ of the IV formulation respectively. Interindividual variability of the AUC is similar after administration by both the IV and oral routes. There is a proportional increase between the AUC and dose. The mean pharmacokinetic parameters were evaluated in blood. After intravenous administration, the terminal half-life averaged 38 hours. Blood clearance was high, approached liver blood flow and averaged 0.72 L/hr/kg (range: 0.32 - 1.26 L/hr/kg), while steady state volume of distribution was large, averaged 21.2 L/kg (range: 7.5 - 39.7 L/kg), and indicated extensive tissue distribution.

Vinorelbine binds extensively to blood cells and especially platelets (70-80%), but less extansively (about 15%) to plasma proteins. There is a significant uptake of NAVELBINE® in lungs, as assessed by pulmonary surgical biopsies showing up to a 300 fold greater concentration than in serum. NAVELBINE® has not been detected in the central nervous system.

NAVELBINE® is mostly metabolised by the CYP 3A4 isoform of the cytochrome P450. All the metabolites have been identified, and none are active except 4-O-deacetylvinorelbine, which is the main metabolite in blood. No sulfo or glucurono conjugates are observed. Renal elimination is low (<20% of the dose) and consists mostly of the parent compound. Biliary excretion is the predominant elimination route of both metabolites and unchanged NAVELBINE®, which is the main recovered compound. The effect of renal dysfunction on NAVELBINE® disposition has not been assessed, however dose reduction in the presence of renal insufficiency is not indicated with

NAVELBINE® due to its low renal elimination.

Vinorelbine is cleared from the circulation primarily by the liver, and therefore elevated blood concentrations may be expected in patients with hepatic impairment. In a Phase I pharmacokinetic study, 6 subjects with severe hepatic impairment were treated with 20 mg/m$^2$ intravenously. Plasma concentrations were elevated compared to historical data from patients with normal hepatic function. Vinorelbine is contraindicated in patients with severe hepatic insufficiency. There is limited experience in patients with mild or moderate hepatic impairment, however available data suggests that dose modification is not required. Haematological toxicity should be closely monitored. A strong relationship was demonstrated between AUC and leucocyte or PMN decreases.

**NAVELBINE® Oral**

Oral vinorelbine has been developed as a line extension of the IV dosage form. Hence the primary objective of the clinical program was to demonstrate bioequivalence between the oral and intravenous formulations on the basis of pharmacokinetic studies. An oral dose of 80 mg/m$^2$ was demonstrated to correspond to 30 mg/m$^2$ of the IV formulation and 60 mg/m$^2$ orally to 25 mg/m$^2$ given by the IV route. Subsequent phase II studies, were conducted to examine the efficacy and tolerance of oral vinorelbine

## Indications

NAVELBINE® is indicated as a single agent or in combination for the treatment of non small cell lung cancer (NSCLC).

## Contraindications

Known hypersensitivity to vinorelbine or other vinca alkaloids.
Disease significantly affecting absorption.
Previous significant surgical resection of stomach or small bowel.
Neutrophil counts < 1500 cells/mm$^3$, or current or recent severe infection due to neutropenia (within 2 weeks).
Pregnancy.
Lactation.
Patients requiring long-term oxygen therapy.
Severe hepatic insufficiency.

## Precautions

### Administration

NAVELBINE® soft capsule should be administered under the supervision of a physician experienced in the use of cancer chemotherapeutic agents. If the patient chews or sucks the capsule by mistake, rinse mouth with water or preferably a normal saline solution.

In the event of the capsule being cut or damaged, the liquid content is an irritant, and so may cause damage if in contact with skin, mucosa or eyes. Damaged capsules should not be swallowed and should be returned to the pharmacy or to the physician in order to be properly destroyed. If any contact occurs, immediate thorough washing with water or preferably with normal saline solution should be undertaken.

If vomiting occurs within a few hours of drug intake, administration of the dose should not be repeated. Prophylactic treatment with metoclopramide or oral antiemetics may reduce the incidence. It is recommended that the capsule be taken with food.

**Myelosuppression**

Neutropenia is dose-limiting. Complete blood counts with differentials should be performed and results reviewed prior to administering each dose of NAVELBINE®. Patients treated with NAVELBINE® should be frequently monitored for myelosuppression both during and after therapy. NAVELBINE® should not be administered to patients with neutrophil counts < 1500 cells/mm$^3$.

Patients developing severe neutropenia should be monitored carefully for evidence of infection and/or fever. If patients present signs or symptoms suggestive of infection, a prompt investigation should be carried out. (See DOSAGE AND ADMINISTRATION for recommended dose adjustments for neutropenia).

NAVELBINE® should be used with extreme caution in patients whose bone marrow reserve may have been compromised by prior irradiation or chemotherapy, or whose marrow function is recovering from the effects of previous chemotherapy (see DOSAGE AND ADMINISTRATION).

During clinical trials where treatment was initiated at a weekly dose of 80 mg/m$^2$ (corresponding to an IV dose of 30 mg/m$^2$ in terms of systemic exposure), febrile neutropenia, in some cases fatal, was encountered in about 15% of patients. Therefore, it is recommended that the starting dose should be 60 mg/m$^2$ and increased to 80 mg/m$^2$ only if the dose is tolerated (see DOSAGE AND ADMINISTRATION).

Since dose-limiting clinical toxicity is the result of depression of the white blood cell count, it is imperative that complete blood counts with differentials be obtained and reviewed on the day of treatment prior to each dose of NAVELBINE®. If the neutrophil count is below 1500 / mm$^3$ and/or the platelet count is between 75,000 and 100,000 / mm$^3$, then treatment should be delayed until recovery.

**General**

Most drug-related adverse events of NAVELBINE® are reversible. If severe adverse events occur, NAVELBINE® should be reduced in dosage or discontinued and appropriate corrective measures taken. Reinstitution of therapy with NAVELBINE® should be carried out with caution and alertness as to possible recurrence of toxicity.

Patients presenting with ischaemic cardiac disease should be carefully monitored (see ADVERSE REACTIONS). Acute shortness of breath and severe bronchospasm have been reported infrequently along with rare cases of interstitial pneumopathy following the administration of NAVELBINE® and other vinca alkaloids, most commonly when the vinca alkaloid was used in combination with mitomycin. These adverse events may require treatment with supplemental oxygen, bronchodilators, and/or corticosteroids, particularly when there is a pre-existing pulmonary dysfunction.

Vinorelbine is contraindicated in patients with severe hepatic insufficiency. There is limited experience in patients with mild or moderate hepatic impairment, however available data suggest that dose modification is not required. Haematological toxicity should be closely monitored.NAVELBINE® should not be given concomitantly with radiotherapy if the treatment field includes the liver. Because of the low level of renal excretion, no dose modification is necessary in patients with renal impairment.

Due to the presence of sorbitol, patients with the rare hereditary problem of fructose intolerance should not take this medicine

**Carcinogenicity/Mutagenicity**

Vinorelbine tartrate has been shown to affect chromosome number and possibly structure in vivo (polyploidy in bone marrow cells from Chinese hamsters and a positive micronucleus test in mice).

It was not mutagenic or cytotoxic in a reverse histidine mutation (Ames) test but showed mutagenic potential in a mouse forward mutation (TK locus) test. Carcinogenicity studies in mice and rats showed no tumourigenic activity at dose levels up to 2.4 mg/m$^2$ given by IV injection every two weeks for 18 months or two years respectively. However, the positive findings in genetic toxicity assays suggest that the drug may have carcinogenic potential at the higher dose level used in humans.

**Effects on fertility**

Adverse effects on the male reproductive system were observed in repeat-dose toxicity studies in animals, including decreased spermatogenesis in rats dosed twice weekly at 2.1 - 7.2 mg/m$^2$ for 13 weeks, reduced prostate/seminal vesicle secretion in rats dosed twice weekly at 3 mg/m$^2$ for 26 weeks, reduced testicular weight in mice dosed at 19 mg/m$^2$/day for three 5-day cycles, and reduced epididymal weight in dogs dosed at 5 mg/m$^2$ for 26 weeks. Vinorelbine tartrate did not affect fertility when administered to male and female rats prior to and during mating; however, the doses used in these studies (9 mg/m$^2$ once weekly or up to 4.2 mg/m$^2$ at 3-day intervals) were lower than the human dose.

**Use in pregnancy**

**Category D**

NAVELBINE® may cause fetal harm if administered to a pregnant woman. When given every three days during organogenesis, vinorelbine tartrate has been shown to be teratogenic in rats and rabbits at doses of 3 and 7.7 mg/m$^2$ respectively. A single 9 mg/m$^2$ dose of vinorelbine tartrate caused embryonic deaths in mice. Doses causing adverse fetal effects in animals were lower than the human dose. There are no studies in pregnant women. If NAVELBINE® is used during pregnancy, or if the patient becomes pregnant while receiving this drug, the patient should be apprised of the potential hazard to the foetus. Women of childbearing potential should be advised to avoid becoming pregnant during therapy with NAVELBINE®.

**Use in lactation**

It is not known whether vinorelbine is excreted in milk of animals or humans. A study in rats showed that growth of the offspring was suppressed when vinorelbine tartrate was administered to lactating dams at 6 mg/m$^2$ every three days. Because many drugs are excreted in human milk, and because of the potential for serious adverse reactions in nursing infants from NAVELBINE®,

it is recommended that nursing be discontinued in women who are receiving therapy with NAVELBINE®.

**Interaction with other drugs**

Acute pulmonary reactions have been reported with NAVELBINE® and other vinca alkaloids used in conjunction with mitomycin. NAVELBINE® should be administered with caution in combination with mitomycin. The combination of NAVELBINE® soft capsules and other drugs with known bone marrow toxicity is likely to exacerbate the myelosuppressive adverse effects. In the absence of specific studies evaluating drug-drug interaction with warfarin, the patient should be cautiously monitored when vinorelbine is given in combination with warfarin. Although the pharmacokinetics of vinorelbine are not influenced by the concurrent administration of cisplatin, the incidence of toxicities, specifically granulocytopenia, with the combination of NAVELBINE® and cisplatin is significantly higher than with single-agent NAVELBINE®.

In studies with rats, the anticoagulant effect of phenindione was potentiated when given in combination with high dose of vinorelbine (30 $mg/m^2$/day for 4 consecutive days or 15 $mg/m^2$/day for 5 consecutive days) but combination treatment with sodium valproate did not cause any increase in anticonvulsant activity. Vinorelbine is metabolised by cytochrome CYP3A4. Although interaction studies have not been performed, it is expected that inhibitors of CYP3A4 such as ketoconazole, itraconazole, ritinovir etc, would result in elevated blood concentrations of vinorelbine. Inducers of CYP3A4 such as rifampicin and phenytoin may reduce concentrations of vinorelbine. Since the magnitude of the inducing or inhibiting effects is unknown, such drug combinations should be avoided.

**Food**

Simultaneous intake of a low fat standard meal does not modify exposure to vinorelbine.

**Paediatric use**

Safety and effectiveness have not been established.

**Geriatric Use**

Clinical experience has not identified differences in responses between the elderly and younger patients, but greater sensitivity of some older individuals cannot be ruled out.

**Adverse Reactions**

The reported incidence of undesirable effects with NAVELBINE® Oral was determined from clinical studies in 210 patients.

**Haematological**

Neutropenia is the dose-limiting toxicity with NAVELBINE®. Neutropenia (Grade 1-2: 24%, Grade 3: 19%, Grade 4: 23.8%) was rapidly reversible and non-cumulative. Grade 4 neutropenia was associated with a fever over 38°C in 2.9% of patients. Further treatment may be given after recovery of the neutrophil count. Infections were observed in 15.2% of patients but were severe in 5.2%. Anaemia was very common but usually mild to moderate (Grade 3: 4.3%, Grade 4: 0.5%). Thrombocytopenia may also occur but was seldom severe (Grade 1 to 2: 12.9%). Dose adjustments are required for haematologic toxicity (see DOSAGE AND ADMINISTRATION).

**Neurological**

Neurosensory disorders were generally limited to loss of deep tendon reflexes (Grades 1 to 2: 12.4%) and infrequently severe. One patient presented partially reversible grade 3 ataxia. Neuromotor disorders were seen in 10.0% of patients (Grade 3: 1.0%). Neuroconstipation was seen in 11.3% of patients (Grades 1 to 2: 10%) and rarely progressed to paralytic ileus (1.4%). One episode of fatal paralytic ileus was reported. Use of prescription laxatives may be appropriate in patients with prior history of constipation and/or who received concomitant treatment with opioid analgesics. Mild to moderate peripheral neuropathy manifested by paraesthesia and loss of deep tendon reflexes (Grade 3: 2.5%, Grade 4: 0.2%) and hyperesthesia have been reported with IV administration. After prolonged treatment, weakness of the lower extremities has also been reported. The effects are dose dependent but usually reversible when treatment is discontinued.

**Gastrointestinal**

Gastrointestinal adverse events occur more commonly with oral vinorelbine than with intravenous administration. Gastrointestinal adverse events reported included: nausea (Grades 1 to 2: 70.5%, Grade 3: 8.6%, Grade 4: 0.5%), vomiting (Grades 1 to 2: 52.9%, Grade 3: 4.3%, Grade 4: 3.3%), diarrhoea (Grades 1 to 2: 41.9%, Grade 3: 2.9%, Grade 4: 2.4%), and anorexia (Grades 1 to 2: 26.7%, Grade 3: 4.8%, Grade 4: 1.0%). Concomitant supportive treatment with metoclopramide or 5HT3 antagonists may reduce the occurrence of nausea and vomiting. Stomatitis usually mild to moderate occurred (Grades 1-2: 8.7%). Oesophagitis was seen in 4.8% of patients (Grade 3: 0.5%). Pancreatitis has been reported very rarely when vinorelbine is given intravenously.

**Other**

Fatigue (Grades 1-2: 19.5%, Grade 3: 6.7%), fever (Grades 1-2: 12.4%), arthralgia including jaw pain, myalgia (Grades 1-2: 9.0%), pain including pain at the tumour site (Grades 1-2: 5.2%) have been experienced by patients receiving NAVELBINE® Oral. Haemorrhagic cystitis and the syndrome of inappropriate ADH secretions were each reported in < 1% of patients given Navelbine® IV. Rare cases of severe hyponatraemia have been reported with Navelbine® IV.
In addition, it cannot be ruled out that the following effects may also be experienced with use of NAVELBINE® Oral, as they have been observed with intravenous administration, and with other vinca alkaloids.

**Cardiovascular**

There have been rare reports of ischemic cardiac disease (angina pectoris, myocardial infarction). In very rare cases, cardiac failure and pulmonary oedema have been reported during treatment with Navelbine® IV, however a causal relationship has not been established.

**Hepatic**

Transient elevations of liver enzymes were reported without clinical symptoms.

**Respiratory**

As with other vinca alkaloids, the intravenous administration of NAVELBINE® has been associated with dyspnea, bronchospasm and rare cases of interstitial pneumopathy in particular in patients treated with NAVELBINE® in combination with mitomycin.

**Dermatological**

Alopecia is usually mild (Grades 1-2: 27.1%) and may appear progressively with extended courses of treatment. Rarely vinca alkaloids may produce generalised cutaneous reactions.

**Adverse Reactions from post-marketing surveillance**

Frequencies are defined as: *very common (>1/10), common (>1/100, <1/10), uncommon (>1/1,000, <1/100), rare (>1/10,000, <1/1,000), very rare (<1/10,000).*

| System organ class (MedDRA classification) | Very common (>10%) | Common (>1% and ≤10%) | Uncommon (>0.1% and ≤1%) |
|---|---|---|---|
| **Blood and lymphatic system disorders** | Neutropenia (grades 1 to 4)<br>Anaemia (grades 1 to 4)<br>Thrombocytopenia (grades 1 to 2) | Anaemia (grade 3) | Anaemia (grade 4) |
| **Gastrointestinal disorders** | Nausea (grades 1 to 2)<br>Vomiting (grades 1 to 2)<br>Diarrhoea (grades 1 to 2)<br>Anorexia (grades 1 to 2) | Nausea (grade 3)<br>Vomiting (grades 3 to 4)<br>Stomatitis (grades 1 to 2)<br>Diarrhoea (grades 3 to 4)<br>Oesophagitis (grades 1 to 2)<br>Anorexia (grade 3) | Nausea (grade 4)<br>Oesophagitis (grade 3)<br>Anorexia (grade 4) |
| **Peripheral autonomic and central nervous system disorders** | Loss of Tendon reflexes (grades 1 to 2) | Neuromotor disorders (grades 1 to 2)<br>Neuro-comstipation (grades 1 to 2)<br>Paralytic ileus | Neuromotor disorders (grade 3)<br>Ataxia (grade 3) |
| **Skin and subcutaneous tissue disorders** | Alopecia (grades 1 to 2) | | |
| **General disorders and administration site conditions** | Fatigue (grades 1 to 2)<br>Fever (grades 1 to 2) | Fatigue (grade 3)<br>Pain (grades 1 to 2) | |
| **Musculoskeletal and connective tissue disorders** | | Arthralgia (grades 1 to 2)<br>Myalgia (grades 1 to 2) | |

**Dosage and Administration**

NAVELBINE® soft capsules must be given strictly via the oral route. They should be swallowed whole with water and should not be chewed or sucked. It is recommended that the capsule be

taken with food.

**Dosage in adults**

**Single agent**

The recommended regimen is:

- **First three administrations:** 60 mg/m$^2$, administered once weekly.
- **Subsequent administrations:** Beyond the third administration, it is recommended to increase the dose of NAVELBINE® soft capsules to 80 mg/m$^2$ once weekly, except in those patients for whom the neutrophil count has dropped once below <500 / mm$^3$ or more than once between 500 and 1000 / mm$^3$, during the first three administrations at 60 mg/m$^2$.

**Dose modifications according to haematological status**

If the neutrophil count is below 1500 / mm$^3$ and/or the platelet count is between 75,000 and 100,000 / mm$^3$, then treatment should be delayed until recovery.

| **Neutrophil count during the first 3 administrations at 60 mg/m$^2$/week** | **Neutrophils >1000** | **Neutrophils ≥ 500 and < 1000 (1 episode)** | **Neutrophils ≥ 500 and < 1000 (2 episodes)** | **Neutrophils <500** |
|---|---|---|---|---|
| **Recommended dose for 4th and subsequent administrations** | **80** | **80** | **60** | **60** |

For any administration planned at the 80 mg/m$^2$/week dose, if the neutrophil count falls below 500 / mm$^3$, the dose must be delayed until recovery and reduced from 80 to 60 mg/m$^2$ per week during the 3 subsequent administrations.

| **Neutrophil count beyond the 4th administration at 80 mg/m$^2$/week** | **Neutrophils >1000** | **Neutrophils ≥ 500 and < 1000 (1 episode)** | **Neutrophils ≥ 500 and < 1000 (2 episodes)** | **Neutrophils <500** |
|---|---|---|---|---|
| **Recommended dose for the next administration** | **80** | | **60** | |

It is possible to re-escalate the dose from 60 to 80 mg/m$^2$/week if the neutrophil count does not drop below 500/mm$^3$, or more than once between 500 and 1000 / mm$^3$, during the three administrations given at the 60 mg/m$^2$ dose.

**Dose modifications for hepatic insufficiency**

Vinorelbine is contraindicated in patients with severe hepatic insufficiency. There is limited experience in patients with mild or moderate hepatic impairment, however available data suggests that dose modification is not required. Haematological toxicity should be closely monitored.

**Combination chemotherapy**

The use of oral vinorelbine in combination regimens has not been extensively studied. However, based on pharmacokinetic studies, the oral dose of 80 mg/m$^2$ was demonstrated to correspond to 30 mg/m$^2$ of the IV form and 60 mg/m$^2$ orally to 25 mg/m$^2$ IV.
In combination regimens, intravenous vinorelbine dosing may be replaced with oral vinorelbine therapy. The recommended dose is 60 mg/m$^2$. The safety of higher doses of oral vinorelbine (eg 80 mg/m$^2$) in combination regimens has not been established.
The following table gives the dose required for appropriate ranges of body surface area (BSA).

| | **60 mg/m$^2$** | **80 mg/m$^2$** |
|---|---|---|
| **BSA (m$^2$)** | **Dose (mg)** | **Dose (mg)** |
| 0.95 to 1 | 60 | 80 |
| 1.05 to 1.14 | 70 | 90 |
| 1.15 to 1.24 | 70 | 100 |
| 1.25 to 1.34 | 80 | 100 |
| 1.35 to 1.44 | 80 | 110 |
| 1.45 to 1.54 | 90 | 120 |
| 1.55 to 1.64 | 100 | 130 |
| 1.65 to 1.74 | 100 | 140 |
| 1.75 to 1.84 | 110 | 140 |
| 1.85 to 1.94 | 110 | 150 |
| ≥ 1.95 | 120 | 160 |

Even patients with a body surface area (BSA) ≥ 2 m$^2$ the dose should never exceed 120 mg per week at 60 mg/m$^2$ and 160 mg per week at 80 mg/m$^2$.
Procedures for proper handling and disposal of anticancer drugs should be used. Several guidelines on this subject have been published.

**Overdose**

There is no known antidote for overdoses of NAVELBINE®. No case of overdosage has been reported with NAVELBINE® oral, however the primary anticipated complications of overdosage would consist of bone marrow suppression and peripheral neurotoxicity. If overdosage occurs, general supportive measures together with appropriate blood transfusions and antibiotics should be instituted as deemed necessary by the physician.

**Presentation**

20 mg soft capsule: light brown soft capsule printed N20,
30 mg soft capsule: pink soft capsule printed N30,
Pack size: 1 capsule

**Shelf life**

Store at 2 to 8° C (Refrigerate. Do not freeze) in the original container.
Protect from light.

### 2.1.2 *Erlotinib (Tarceva®)*

**Description**

TARCEVA (erlotinib) is a Human Epidermal Growth Factor Receptor Type 1/Epidermal Growth Factor Receptor (HER1/EGFR) tyrosine kinase inhibitor. Erlotinib is a quinazolinamine with the chemical name N-(3-ethynylphenyl)-6,7-bis(2-methoxyethoxy)-4-quinazolinamine. TARCEVA contains erlotinib as the hydrochloride salt that has the following structural formula:

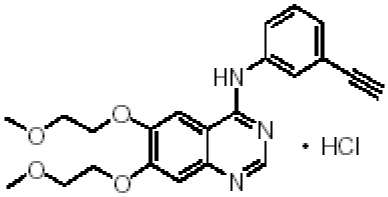

Erlotinib hydrochloride has the molecular formula $C_{22}H_{23}N_3O_4$.HCl and a molecular weight of 429.90. The molecule has a $pK_a$ of 5.42 at 25°C. Erlotinib hydrochloride is very slightly soluble in water, slightly soluble in methanol and practically insoluble in acetonitrile, acetone, ethyl acetate and hexane.

Aqueous solubility of erlotinib hydrochloride is dependent on pH with increased solubility at a pH of less than 5 due to protonation of the secondary amine. Over the pH range of 1.4 to 9.6, maximal solubility of approximately 0.4 mg/mL occurs at a pH of approximately 2.

TARCEVA tablets are available in three dosage strengths containing erlotinib hydrochloride (27.3 mg, 109.3 mg and 163.9 mg) equivalent to 25 mg, 100 mg and 150 mg erlotinib and the following inactive ingredients: lactose monohydrate, hypromellose, hydroxypropyl cellulose, magnesium stearate, microcrystalline cellulose, sodium starch glycolate, sodium lauryl sulfate and titanium dioxide. The tablets also contain trace amounts of color additives, including FD&C Yellow #6 (25 mg only) for product identification.

**Clinical Pharmacology**

**Mechanism of Action and Pharmacodynamics**

The mechanism of clinical antitumor action of erlotinib is not fully characterized. Erlotinib inhibits the intracellular phosphorylation of tyrosine kinase associated with the epidermal growth factor receptor (EGFR). Specificity of inhibition with regard to other tyrosine kinase receptors has not been fully characterized. EGFR is expressed on the cell surface of normal cells and cancer cells.

**Pharmacokinetics**

Erlotinib is about 60% absorbed after oral administration and its bioavailability is substantially increased by food to almost 100%. Its half-life is about 36 hours and it is cleared predominantly by CYP3A4 metabolism and to a lesser extent by CYP1A2.

**Absorption and Distribution**

Bioavailability of erlotinib following a 150 mg oral dose of TARCEVA is about 60% and peak plasma levels occur 4 hrs after dosing. Food increases bioavailability substantially, to almost 100%. Following absorption, erlotinib is approximately 93% protein bound to albumin and alpha-1 acid glycoprotein (AAG). Erlotinib has an apparent volume of distribution of 232 liters.

**Metabolism and Elimination**

In vitro assays of cytochrome P450 metabolism showed that erlotinib is metabolized primarily by CYP3A4 and to a lesser extent by CYP1A2, and the extrahepatic isoform CYP1A1. Following a 100 mg oral dose, 91% of the dose was recovered: 83% in feces (1% of the dose as intact parent)

and 8% in urine (0.3% of the dose as intact parent). A population pharmacokinetic analysis in 591 patients receiving single-agent TARCEVA showed a median half-life of 36.2 hours. Time to reach steady state plasma concentration would therefore be 7 — 8 days. No significant relationships of clearance to covariates of patient age, body weight or gender were observed. Smokers had a 24% higher rate of erlotinib clearance. A second population pharmacokinetic analysis was conducted that incorporated erlotinib data from 204 pancreatic cancer patients who received erlotinib plus gemcitabine. This analysis demonstrated that covariates affecting erlotinib clearance in patients from the pancreatic study were very similar to those seen in the prior single-agent pharmacokinetic analysis. No new covariate effects were identified. Co-administration of gemcitabine had no effect on erlotinib plasma clearance.

**Special Populations**

**Patients with Hepatic Impairment**

Erlotinib is cleared predominantly by the liver. No data are currently available regarding the influence of hepatic dysfunction and/or hepatic metastases on the pharmacokinetics of erlotinib.

**Patients with Renal Impairment**

Less than 9% of a single dose is excreted in the urine. No clinical studies have been conducted in patients with compromised renal function.

**Interactions**

Erlotinib is metabolized predominantly by CYP3A4, and inhibitors of CYP3A4 would be expected to increase exposure. Co-treatment with the potent CYP3A4 inhibitor ketoconazole increased erlotinib AUC by 2/3. Pre- or co-treatment with the CYP3A4 inducer rifampicin increased erlotinib clearance by 3-fold and reduced AUC by 2/3. In a Phase Ib study, there were no significant effects of gemcitabine on the pharmacokinetics of erlotinib nor were there significant effects of erlotinib on the pharmacokinetics of gemcitabine.

## Indications

**Non-Small Cell Lung Cancer**

TARCEVA monotherapy is indicated for the treatment of patients with locally advanced or metastatic non-small cell lung cancer after failure of at least one prior chemotherapy regimen. Results from two, multicenter, placebo-controlled, randomized, Phase 3 trials conducted in first-line patients with locally advanced or metastatic NSCLC showed no clinical benefit with the concurrent administration of TARCEVA with platinum-based chemotherapy [carboplatin and paclitaxel or gemcitabine and cisplatin] and its use is not recommended in that setting.

**Pancreatic Cancer**

TARCEVA in combination with gemcitabine is indicated for the first-line treatment of patients with locally advanced, unresectable or metastatic pancreatic cancer.

## Dosage and Administration

### Non-Small Cell Lung Cancer

The recommended daily dose of TARCEVA is 150 mg taken at least one hour before or two hours after the ingestion of food. Treatment should continue until disease progression or unacceptable toxicity occurs. There is no evidence that treatment beyond progression is beneficial.

### Pancreatic Cancer

The recommended daily dose of TARCEVA is 100 mg taken at least one hour before or two hours after the ingestion of food, in combination with gemcitabine (see the gemcitabine package insert). Treatment should continue until disease progression or unacceptable toxicity occurs.

### Dose Modifications

In patients who develop an acute onset of new or progressive pulmonary symptoms, such as dyspnea, cough or fever, treatment with TARCEVA should be interrupted pending diagnostic evaluation. If ILD is diagnosed, TARCEVA should be discontinued and appropriate treatment instituted as necessary.

Diarrhea can usually be managed with loperamide. Patients with severe diarrhea who are unresponsive to loperamide or who become dehydrated may require dose reduction or temporary interruption of therapy. Patients with severe skin reactions may also require dose reduction or temporary interruption of therapy.

When dose reduction is necessary, the TARCEVA dose should be reduced in 50 mg decrements. In patients who are being concomitantly treated with a strong CYP3A4 inhibitor such as, but not limited to, atazanavir, clarithromycin, indinavir, itraconazole, ketoconazole, nefazodone, nelfinavir, ritonavir, saquinavir, telithromycin, troleandomycin (TAO), or voriconazole, a dose reduction should be considered should severe adverse reactions occur.

Pre-treatment with the CYP3A4 inducer rifampicin decreased erlotinib AUC by about 2/3. Alternate treatments lacking CYP3A4 inducing activity should be considered. If an alternative treatment is unavailable, a TARCEVA dose greater than 150 mg should be considered. If the TARCEVA dose is adjusted upward, the dose will need to be reduced upon discontinuation of rifampicin or other inducers. Other CYP3A4 inducers include, but are not limited to rifabutin, rifapentine, phenytoin, carbamazepine, phenobarbital and St. John's Wort. These too should be avoided if possible.

Erlotinib is eliminated by hepatic metabolism and biliary excretion. Therefore, caution should be used when administering TARCEVA to patients with hepatic impairment. Dose reduction or interruption of TARCEVA should be considered should severe adverse reactions occur.

### Side Effects

Safety evaluation of TARCEVA is based on 856 cancer patients who received TARCEVA as monotherapy, 308 patients who received TARCEVA 100 or 150 mg plus gemcitabine, and 1228 patients who received TARCEVA concurrently with other chemotherapies.

**Non-Small Cell Lung Cancer**
Adverse events, regardless of causality, that occurred in at least 10% of patients treated with single-agent TARCEVA at 150 mg and at least 3% more often than in the placebo group in the randomized trial of patients with NSCLC are summarized by NCI-CTC (version 2.0) Grade in the table below.

The most common adverse reactions in patients receiving single-agent TARCEVA 150 mg were rash and diarrhea. Grade 3/4 rash and diarrhea occurred in 9% and 6%, respectively, in TARCEVA-treated patients. Rash and diarrhea each resulted in study discontinuation in 1% of TARCEVA-treated patients. Six percent and 1% of patients needed dose reduction for rash and diarrhea, respectively. The median time to onset of rash was 8 days, and the median time to onset of diarrhea was 12 days.

**Adverse Events Occurring in ≥10% of Single-Agent TARCEVA-treated Non-Small Cell Lung Cancer Patients (2:1 Randomization of TARCEVA to Placebo)**

| | **TARCEVA 150 mg N = 485** | | | **Placebo N = 242** | | |
|---|---|---|---|---|---|---|
| **NCI CTC Grade** | **Any Grade** | **Grade 3** | **Grade 4** | **Any Grade** | **Grade 3** | **Grade 4** |
| **MedDRA Preferred Term** | % | % | % | % | % | % |
| Rash | 75 | 8 | <1 | 17 | 0 | 0 |
| Diarrhea | 54 | 6 | <1 | 18 | <1 | 0 |
| Anorexia | 52 | 8 | 1 | 38 | 5 | <1 |
| Fatigue | 52 | 14 | 4 | 45 | 16 | 4 |
| Dyspnea | 41 | 17 | 11 | 35 | 15 | 11 |
| Cough | 33 | 4 | 0 | 29 | 2 | 0 |
| Nausea | 33 | 3 | 0 | 24 | 2 | 0 |
| Infection | 24 | 4 | 0 | 15 | 2 | 0 |
| Vomiting | 23 | 2 | <1 | 19 | 2 | 0 |
| Stomatitis | 17 | <1 | 0 | 3 | 0 | 0 |
| Pruritus | 13 | <1 | 0 | 5 | 0 | 0 |
| Dry skin | 12 | 0 | 0 | 4 | 0 | 0 |
| Conjunctivitis | 12 | <1 | 0 | 2 | <1 | 0 |
| Keratoconjunctivitis sicca | 12 | 0 | 0 | 3 | 0 | 0 |
| Abdominal pain | 11 | 2 | <1 | 7 | 1 | <1 |

Liver function test abnormalities (including elevated alanine aminotransferase (ALT), aspartate aminotransferase (AST) and bilirubin) were observed in patients receiving single-agent TARCEVA 150 mg. These elevations were mainly transient or associated with liver metastases. Grade 2 (>2.5 — 5.0 x ULN) ALT elevations occurred in 4% and <1% of TARCEVA and placebo treated patients, respectively. Grade 3 (>5.0 — 20.0 x ULN) elevations were not observed in TARCEVA-treated patients. Dose reduction or interruption of TARCEVA should be considered if changes in liver function are severe.

**Pancreatic Cancer**

Adverse events, regardless of causality, that occurred in at least 10% of patients treated with TARCEVA 100 mg plus gemcitabine in the randomized trial of patients with pancreatic cancer are summarized by NCI-CTC (version 2.0) Grade in the table below. The most common adverse reactions in pancreatic cancer patients receiving TARCEVA 100 mg plus gemcitabine were fatigue, rash, nausea, anorexia and diarrhea. In the TARCEVA plus gemcitabine arm, Grade 3/4 rash and diarrhea were each reported in 5% of TARCEVA plus gemcitabine-treated patients. The median time to onset of rash and diarrhea was 10 days and 15 days, respectively. Rash and diarrhea each resulted in dose reductions in 2% of patients, and resulted in study discontinuation in up to 1% of patients receiving TARCEVA plus gemcitabine. The 150 mg cohort was associated with a higher rate of certain class-specific adverse reactions including rash and required more frequent dose reduction or interruption.

**Adverse Events occurring in ≥10% of TARCEVA-treated Pancreatic Cancer Patients: 100 mg cohort**

| | TARCEVA + Gemcitabine 1000 mg/m² IV N=259 | | | Placebo + Gemcitabine 1000 mg/m² IV N=256 | | |
|---|---|---|---|---|---|---|
| **NCI CTC Grade** | **Any Grade** | **Grade 3** | **Grade 4** | **Any Grade** | **Grade 3** | **Grade 4** |
| **MedDRA Preferred Term** | **%** | **%** | **%** | **%** | **%** | **%** |
| Fatigue | 73 | 14 | 2 | 70 | 13 | 2 |
| Rash | 69 | 5 | 0 | 30 | 1 | 0 |
| Nausea | 60 | 7 | 0 | 58 | 7 | 0 |
| Anorexia | 52 | 6 | <1 | 52 | 5 | <1 |
| Diarrhea | 48 | 5 | <1 | 36 | 2 | 0 |
| Abdominal pain | 46 | 9 | <1 | 45 | 12 | <1 |
| Vomiting | 42 | 7 | <1 | 41 | 4 | <1 |
| Weight decreased | 39 | 2 | 0 | 29 | <1 | 0 |
| Infection* | 39 | 13 | 3 | 30 | 9 | 2 |
| Edema | 37 | 3 | <1 | 36 | 2 | <1 |
| Pyrexia | 36 | 3 | 0 | 30 | 4 | 0 |
| Constipation | 31 | 3 | 1 | 34 | 5 | 1 |
| Bone pain | 25 | 4 | <1 | 23 | 2 | 0 |
| Dyspnea | 24 | 5 | <1 | 23 | 5 | 0 |
| Stomatitis | 22 | <1 | 0 | 12 | 0 | 0 |
| Myalgia | 21 | 1 | 0 | 20 | <1 | 0 |
| Depression | 19 | 2 | 0 | 14 | <1 | 0 |
| Dyspepsia | 17 | <1 | 0 | 13 | <1 | 0 |

| | | | | | | |
|---|---|---|---|---|---|---|
| Cough | 16 | 0 | 0 | 11 | 0 | 0 |
| Dizziness | 15 | <1 | 0 | 13 | 0 | <1 |
| Headache | 15 | <1 | 0 | 10 | 0 | 0 |
| Insomnia | 15 | <1 | 0 | 16 | <1 | 0 |
| Alopecia | 14 | 0 | 0 | 11 | 0 | 0 |
| Anxiety | 13 | 1 | 0 | 11 | <1 | 0 |
| Neuropathy | 13 | 1 | <1 | 10 | <1 | 0 |
| Flatulence | 13 | 0 | 0 | 9 | <1 | 0 |
| Rigors | 12 | 0 | 0 | 9 | 0 | 0 |

*Includes all MedDRA preferred terms in the Infections and Infestations System Organ Class.

In the pancreatic carcinoma trial, 10 patients in the TARCEVA/gemcitabine group developed deep venous thrombosis (incidence: 3.9%). In comparison, 3 patients in the placebo/gemcitabine group developed deep venous thrombosis (incidence 1.2%). The overall incidence of grade 3 or 4 thrombotic events, including deep venous thrombosis, was similar in the two treatment arms: 11% for TARCEVA plus gemcitabine and 9% for placebo plus gemcitabine.
No differences in Grade 3 or Grade 4 hematologic laboratory toxicities were detected between the TARCEVA plus gemcitabine group compared to the placebo plus gemcitabine group.
Severe adverse events (≥grade 3 NCI CTC) in the TARCEVA plus gemcitabine group with incidences < 5% included syncope, arrhythmias, ileus, pancreatitis, hemolytic anemia including microangiopathic hemolytic anemia with thrombocytopenia, myocardial infarction/ischemia, cerebrovascular accidents including cerebral hemorrhage, and renal insufficiency.

Liver function test abnormalities (including elevated alanine aminotransferase (ALT), aspartate aminotransferase (AST) and bilirubin) have been observed following the administration of TARCEVA plus gemcitabine in patients with pancreatic cancer. The table below displays the most severe NCI-CTC grade of liver function abnormalities that developed. Dose reduction or interruption of TARCEVA should be considered if changes in liver function are severe.

**Liver Function Test Abnormalities (most severe NCI-CTC grade) in Pancreatic Cancer Patients: 100 mg Cohort**

| | **TARCEVA + Gemcitabine 1000 mg/m2 IV N = 259** | | | **Placebo + Gemcitabine 1000 mg/m2 IV N = 256** | | |
|---|---|---|---|---|---|---|
| **NCI CTC Grade** | **Grade 2** | **Grade 3** | **Grade 4** | **Grade 2** | **Grade 3** | **Grade 4** |
| Bilirubin | 17% | 10% | <1% | 11% | 10% | 3% |
| ALT | 31% | 13% | <1% | 22% | 9% | 0% |
| AST | 24% | 10% | <1% | 19% | 9% | 0% |

**NSCLC and Pancreatic Cancer Indications**
During the NSCLC and the combination pancreatic cancer trials, infrequent cases of gastrointestinal bleeding have been reported, some associated with concomitant warfarin or NSAID administration. These adverse events were reported as peptic ulcer bleeding (gastritis,

gastroduodenal ulcers), hematemesis, hematochezia, melena and hemorrhage from possible colitis. Cases of Grade 1 epistaxis were also reported in both the single-agent NSCLC and the pancreatic cancer clinical trials.

NCI-CTC Grade 3 conjunctivitis and keratitis have been reported infrequently in patients receiving TARCEVA therapy in the NSCLC and pancreatic cancer clinical trials. Corneal ulcerations may also occur.

In general, no notable differences in the safety of TARCEVA monotherapy or in combination with gemcitabine could be discerned between females or males and between patients younger or older than the age of 65 years. The safety of TARCEVA appears similar in Caucasian and Asian patients.

**Drug Interactions**

Co-treatment with the potent CYP3A4 inhibitor ketoconazole increases erlotinib AUC by 2/3. Caution should be used when administering or taking TARCEVA with ketoconazole and other strong CYP3A4 inhibitors such as, but not limited to, atazanavir, clarithromycin, indinavir, itraconazole, nefazodone, nelfinavir, ritonavir, saquinavir, telithromycin, troleandomycin (TAO), and voriconazole. Pre-treatment with the CYP3A4 inducer rifampicin decreased erlotinib AUC by about 2/3. Alternate treatments lacking CYP3A4 inducing activity should be considered. If an alternative treatment is unavailable, a TARCEVA dose greater than 150 mg should be considered for NSCLC patients, and greater than 100 mg considered for pancreatic cancer patients. If the TARCEVA dose is adjusted upward, the dose will need to be reduced upon discontinuation of rifampicin or other inducers. Other CYP3A4 inducers include, but are not limited to, rifabutin, rifapentine, phenytoin, carbamazepine, phenobarbital and St. John's Wort

## Warnings

**Pulmonary Toxicity**

There have been infrequent reports of serious Interstitial Lung Disease (ILD)-like events, including fatalities, in patients receiving TARCEVA for treatment of NSCLC, pancreatic cancer or other advanced solid tumors. In the randomized single-agent NSCLC study, the incidence of ILD-like events (0.8%) was the same in both the placebo and TARCEVA groups. In the pancreatic cancer study - in combination with gemcitabine, the incidence of ILD-like events was 2.5% in the TARCEVA plus gemcitabine group vs. 0.4% in the placebo plus gemcitabine group.

The overall incidence of ILD-like events in approximately 4900 TARCEVA-treated patients from all studies (including uncontrolled studies and studies with concurrent chemotherapy) was approximately 0.7%. Reported diagnoses in patients suspected of having ILD-like events included pneumonitis, radiation pneumonitis, hypersensitivity pneumonitis, interstitial pneumonia, interstitial lung disease, obliterative bronchiolitis, pulmonary fibrosis, Acute Respiratory Distress Syndrome and lung infiltration. Symptoms started from 5 days to more than 9 months (median 39 days) after initiating TARCEVA therapy. In the lung cancer trials most of the cases were associated with confounding or contributing factors such as concomitant/prior chemotherapy, prior radiotherapy, pre-existing parenchymal lung disease, metastatic lung disease, or pulmonary infections.

In the event of an acute onset of new or progressive, unexplained pulmonary symptoms such as dyspnea, cough, and fever, TARCEVA therapy should be interrupted pending diagnostic evaluation. If ILD is diagnosed, TARCEVA should be discontinued and appropriate treatment instituted as needed

**Myocardial infarction/ischemia:**
In the pancreatic carcinoma trial, six patients (incidence of 2.3%) in the TARCEVA/gemcitabine group developed myocardial infarction/ischemia. One of these patients died due to myocardial infarction. In comparison, 3 patients in the placebo/gemcitabine group developed myocardial infarction (incidence 1.2%) and one died due to myocardial infarction.

**Cerebrovascular accident:**
In the pancreatic carcinoma trial, six patients in the TARCEVA/gemcitabine group developed cerebrovascular accidents (incidence: 2.3%) One of these was hemorrhagic and was the only fatal event. In comparison, in the placebo/gemcitabine group there were no cerebrovascular accidents.

**Microangiopathic Hemolytic Anemia with Thrombocytopenia:**
In the pancreatic carcinoma trial, two patients in the TARCEVA/gemcitabine group developed microangiopathic hemolytic anemia with thrombocytopenia (incidence: 0.8%). Both patients received TARCEVA and gemcitabine concurrently. In comparison, in the placebo/gemcitabine group there were no cases of microangiopathic hemolytic anemia with thrombocytopenia.

**Pregnancy**
Erlotinib has been shown to cause maternal toxicity with associated embryo/fetal lethality and abortion in rabbits when given at doses that result in plasma drug concentrations of approximately 3 times those in humans (AUCs at 150 mg daily dose). When given during the period of organogenesis to achieve plasma drug concentrations approximately equal to those in humans, based on AUC, there was no increased incidence of embryo/fetal lethality or abortion in rabbits or rats. However, female rats treated with 30 mg/m$^2$/day or 60 mg/m$^2$/day (0.3 or 0.7 times the clinical dose, on a mg/m$^2$ basis) of erlotinib prior to mating through the first week of pregnancy had an increase in early resorptions that resulted in a decrease in the number of live fetuses. No teratogenic effects were observed in rabbits or rats. There are no adequate and well-controlled studies in pregnant women using TARCEVA. Women of childbearing potential should be advised to avoid pregnancy while on TARCEVA. Adequate contraceptive methods should be used during therapy, and for at least 2 weeks after completing therapy. Treatment should only be continued in pregnant women if the potential benefit to the mother outweighs the risk to the fetus. If TARCEVA is used during pregnancy, the patient should be apprised of the potential hazard to the fetus or potential risk for loss of the pregnancy.

## Precautions

### Hepatotoxicity
Asymptomatic increases in liver transaminases have been observed in TARCEVA treated patients; therefore, periodic liver function testing (transaminases, bilirubin, and alkaline phosphatase) should be considered. Dose reduction or interruption of TARCEVA should be considered if changes in liver function are severe.

**Patients with Hepatic Impairment**
*In vitro* and *in vivo* evidence suggest that erlotinib is cleared primarily by the liver. Therefore, erlotinib exposure may be increased in patients with hepatic dysfunction

**Elevated International Normalized Ratio and Potential Bleeding**
International Normalized Ratio (INR) elevations and infrequent reports of bleeding events including gastrointestinal and non-gastrointestinal bleedings have been reported in clinical studies, some associated with concomitant warfarin administration. Patients taking warfarin or other coumarin-derivative anticoagulants should be monitored regularly for changes in prothrombin time or INR.

**Carcinogenesis, Mutagenesis, Impairment of Fertility**
Erlotinib has not been tested for carcinogenicity. Erlotinib has been tested for genotoxicity in a series of *in vitro* assays (bacterial mutation, human lymphocyte chromosome aberration, and mammalian cell mutation) and an *in vivo* mouse bone marrow micronucleus test and did not cause genetic damage. Erlotinib did not impair fertility in either male or female rats.

**Nursing Mothers**
It is not known whether erlotinib is excreted in human milk. Because many drugs are excreted in human milk and because the effects of TARCEVA on infants have not been studied, women should be advised against breast-feeding while receiving TARCEVA therapy.

**Pediatric Use**
The safety and effectiveness of TARCEVA in pediatric patients have not been studied.

**Geriatric Use**
Of the total number of patients participating in the randomized NSCLC trial, 62% were less than 65 years of age, and 38% of patients were aged 65 years or older. The survival benefit was maintained across both age groups. In the pancreatic cancer study, 53% of patients were younger than 65 years of age and 47% were 65 years of age or older. No meaningful differences in safety or pharmacokinetics were observed between younger and older patients in either study. Therefore, no dosage adjustments are recommended in elderly patients.

## 2.2 NSCLC

Non-small cell lung cancer is the commonest cause of cancer mortality in Singapore. Up to 70% of patients are diagnosed with advanced stage IIIB and IV disease at presentation and significant proportion of patients with resected disease eventually relapse and die of disease. In Asia, the number of patients with lung cancer is expected to grow given the increasing prevalence of smoking in the local populations. Conventional palliative chemotherapy has reached a plateau in survival benefit and there is a need for novel agents for treatment of this disease. The introduction of EGFR TK-I into the treatment of NSCLC gives additional improvement in survival in a select percentage of patients only. There is therefore a clear need for new approaches in treating metastatic NSCLC patients in the salvage setting as all "classical" cytotoxic or targeted drugs provide the same range of efficacy.

Erlotinib is currently approved for use in the salvage setting as monotherapy for non-small cell lung carcinoma. However, response rates are around 10% in a large phase III study (1). Equivalent response rates are achieved using conventional cytotoxics in the salvage setting (2-4). Combining an oral cytotoxic with an oral targeted therapy provides the convenience and cost-savings of oral therapy with the potential of increasing response rates.

## 2.3 Rational

Additive or supradditive activity of an EGFR TK-I with vinorelbine has been demonstrated in-vitro (5). Clinical synergism has also been described between gefitinib and vinorelbine in NSCLC (6). The use of cytotoxics in a metronomic schedule has not been well investigated in the clinical setting despite emerging pre-clinical data. Using an established oral cytotoxic such as oral vinorelbine in a metronomic dose-schedule is attractive due to the oral route of administration. Preclinical studies have shown that by using cytotoxics in a low-dose protracted manner, endothelial cells are preferentially affected via inhibition of proliferation and induction of apoptosis (7). In addition to this anti-angiogenic mechanism, an anti-vasculogenic process may also be involved that acts by reducing circulating endothelial progenitor mobilization and viability (8). Moreover, it has also been shown that tumours that were selected for high levels of acquired resistance to cytotoxics can be induced to respond by using metronomic doses of chemotherapy (9, 10).

Continuous administration of metronomic oral vinorelbine, given three times a week, has been reported as feasible and well tolerated at doses up to 180 mg total dose per week. Early results showed activity against refractory solid tumors such as renal cancer, NSCLC, ovarian cancer, prostate cancer, unknown primary and Kaposi sarcoma (Briasoulis et al EORTC-NCI-AACR 2006). Eighty patients (39 female and 41 male, median age 58, median PS 1) were treated in this study with a variety of progressive refractory solid cancers and were treated at the dose range of 20 mg (16 pts), 30 mg (17 pts), 40 mg (26 pts), 50 mg (13 pts), 60 mg (6 pts) and 70 mg (2 pts) TTW. Dose limiting toxicity occurred in 2 patients at the 60 mg dose level (leucopenia grade 4 on the 14$^{th}$ week of treatment and epistaxis on the 9$^{th}$ week treatment). Median duration of treatment was 19 weeks (range 4 to 85+ weeks) and corresponded to time to progression, since no withdrawals from therapy were due to toxicity. Objective tumor response was documented in 8 among 52 patients in renal cancer, NSCLC, ovarian cancer, prostate cancer, unknown primary and Kaposi sarcoma while 32% of treated patients had disease control for at least 6 months. A similar metronomic dosing schedule was also shown to be safe and feasible up to 50 mg three times a week (11).

However, determining the optimal metronomic dose of a cytotoxic agent is challenging as optimal antiangiogenic activity would likely be below the maximum tolerated dose (MTD). Using levels of circulating endothelial cells (CECs) and circulating endothelial progenitor cells (CEPs) has been shown to good surrogate

pharmacodynamic marker to monitor antiangiogenic activity of a drug (13).

This phase I study combines erlotinib and oral vinorelbine on two different schedules. The conventional schedule vinorelbine (CSV) aims to determine the MTD of conventional schedule of oral vinorelbine given on days 1 and 8 every 21 days plus daily erlotinib and the metronomic schedule vinorelbine (MSV) aims to determine the optimal metronomic dose of vinorelbine given 3 times a week plus daily erlotinib.

The objectives are:

a) To assess whether combination of oral vinorelbine and erlotinib is safe and feasible
b) To determine dosing of oral vinorelbine on a metronomic schedule leading to anti-angiogenesis as demonstrated by CEP/CEC assays

## 3. PATIENT SELECTION

### 3.1 Eligibility Criteria

3.1.1 Patients must have histologically or cytologically confirmed *NSCLC*

3.1.2 *At least one or two prior lines of chemotherapy for metastatic disease or locally advanced unresectable disease*. There should be at least 4 weeks since prior chemotherapy or radiation therapy or 6 weeks if the last regimen included BCNU or mitomycin C

3.1.3 Age $\geq$ 21 years.

3.1.4 ECOG performance status <2 (Karnofsky >60%, see Appendix A).

3.1.5 Life expectancy of greater than 3 months

3.1.6 Patients must have normal organ and marrow function as defined below:

| | |
|---|---|
| – leukocytes | >3,000/mcL |
| – absolute neutrophil count | >1,500/mcL |
| – platelets | >100,000/mcL |
| – total bilirubin | within normal institutional limits |
| – AST(SGOT)/ALT(SGPT) | <2.5 X institutional upper limit of normal |
| – creatinine | within normal institutional limits |
| OR | |
| – creatinine clearance | >60 mL/min/1.73 $m^2$ for patients with creatinine levels above institutional normal. |

3.1.7 The effects of Oral Vinorelbine on the developing human fetus are unknown. For this reason and because vinca alkaloids as well as other therapeutic agents used in this trial are known to be teratogenic, women of

child-bearing potential and men must agree to use adequate contraception (hormonal or barrier method of birth control; abstinence) prior to study entry and for the duration of study participation. Should a woman become pregnant or suspect she is pregnant while participating in this study, she should inform her treating physician immediately.

3.1.8 Ability to understand and the willingness to sign a written informed consent document.

## 3.2 **Exclusion Criteria**

3.2.1 Patients who have had chemotherapy or radiotherapy within 4 weeks (6 weeks for nitrosoureas or mitomycin C) prior to entering the study or those who have not recovered from adverse events due to agents administered more than 4 weeks earlier.

3.2.2 Patients may not be receiving any other investigational agents.

3.2.3 Patients who have received previous vinorelbine or oral EGFR tyrosine kinase inhibitors

3.2.4 Patients with *progressive* brain metastases should be excluded from this clinical trial because of their poor prognosis and because they often develop progressive neurologic dysfunction that would confound the evaluation of neurologic and other adverse events. *However patients are eligible if they have brain metastases that have been treated with whole brain radiotherapy and are stable and not on corticosteroids.*

3.2.5 History of allergic reactions attributed to compounds of similar chemical or biologic composition to Oral Vinorelbine or other agents used in study.

3.2.6 Prior and / or concomitant treatment with drugs known to induce or inhibit cytochrome P450 3A4, CYP1A1 & CYP1A2 : phenytoin, carbamazepine, barbiturates, rifampicine, imidazole antifungals (such as ketoconazole, fluconazole, itraconazole, metronidazole), omeprazole and ritonavir *within 1 week of starting protocol treatment.*

3.2.7 Significant malabsorption syndrome or disease affecting the gastro-intestinal tract function

3.2.8 Uncontrolled intercurrent illness including, but not limited to, ongoing or active infection, symptomatic congestive heart failure, unstable angina pectoris, cardiac arrhythmia, or psychiatric illness/social situations that would limit compliance with study requirements.

3.2.9 Pregnancy or breast feeding or women of child-bearing potential not using effective contraception.

3.2.10 HIV-positive patients on combination antiretroviral therapy are ineligible because of the potential for pharmacokinetic interactions with Oral Vinorelbine. In addition, these patients are at increased risk of lethal infections when treated with marrow-suppressive therapy. Appropriate studies will be undertaken in patients receiving combination antiretroviral therapy when indicated.

3.2.11 History of organ allograft

3.2.12 Patients with evidence or history of bleeding diasthesis or coagulopathy

3.2.13 Serious, non-healing wound, ulcer, or bone fracture

3.2.14 Because of interaction risk on CYP3A4, patients with concomitant treatments with vitamin K antagonists such as phenprocoumon or warfarin

### 3.3 Inclusion of Women and Minorities

Both men and women of all races and ethnic groups are eligible for this trial.

## 4. REGISTRATION PROCEDURES

*N.A*

## 5. TREATMENT PLAN

### 5.1 Agent Administration

This is a Phase I Dose-Finding Study examining the combination of vinorelbine/erlotinib. The MTD approach will be used for both phase I schedules as shown in table below. Treatment will be administered on an outpatient basis. Oral vinorelbine will be started according to two different schedules (see tables below) 2 days before starting erlotinib. A “ping-pong” phase 1 design is employed allowing each schedule to accrue sequentially at each dose level (see figure below). This design expedites accrual without compromising safety and monitoring. All accrual will start at dose level 1. When CSV level 1 has completed accrual, MSV level 1 will start accrual while waiting for CSV level 1 patients to complete their first cycle of treatment. When MSV level 1 has completed accrual, CSV level 2 will begin and the cycle repeats itself till dose level 5 or unless DLT is reached for one schedule, and so on till DLT or dose level 5 reached. Once the MTD in one schedule is reached before level 5, the other schedule will continue till the MTD or level 5 is reached

| Dose Escalation Schedule | | | |
|---|---|---|---|
| | Dose | | |
| | **CSV** | **MSV** | **Dose Level** |
| | **Vinorelbine (mg/m$^2$ on D1,D8 every 21 days)** | **Vinorelbine (mg/week)** | |
| **Erlotinib 100 mg** (start 2 days after vinorelbine for cycle 1) | 30 | 80 | Level -1 |
| | 40 | 100 | Level 1 |
| | 50 | 120 | Level 2 |
| | 60 | 140 | Level 3 |
| | 70 | 160 | Level 4 |
| | 80 | 180 | Level 5 |
| **Erlotinib 150 mg** (only in the absence of DLT at dose level 5 of vinorelbine) | No DLT at dose level 5 of vinorelbine (80 mg/m2) | No DLT at dose level 5 of vinorelbine (180 mg) | |

Escalation of Erlotinib dose to 150 mg OM will only start if level 5 oral vinorelbine is reached without dose-limiting toxicities with Erlotinib 100 mg OM in each schedule.

The optimal anti-angiogenic dose of oral vinorelbine given in metronomic schedule will be determined from the Circulating Endothelial Cells/Circulating Endothelial Progenitors (CEC/CEP) assays correlations.
Efficacy will be assessed every 2 cycles during maintenance treatment. Each cycle will last 21 days with a window period of 7 days. Patients on the conventional schedule of vinorelbine who respond or have stable disease, vinorelbine will be stopped after 6 cycles and erlotinib continued. Patients on the metronomic schedule of vinorelbine who respond will continue on both vinorelbine and erlotinib.

Reported adverse events and potential risks for Oral Vinorelbine and Erlotinib are described in Section 5.2. Appropriate dose modifications for Oral Vinorelbine and Erlotinib are described in Section 6. No investigational or commercial agents or therapies other than those described below may be administered with the intent to treat the patient's malignancy.

**REGIMEN DESCRIPTION**

| Agent | Premedications; Precautions | Dose | Route | Schedule | Cycle Length |
|---|---|---|---|---|---|
| Vinorelbine | Both erlotinib and vinorelbine will be given after prophylactic administration of antiemetics (preferably 5-HT3 antagonist) on Day 1 (except for Cycle 1) and on Day 8 of each cycle | Dose as appropriate for assigned dose level | Oral | **Conventional Schedule**: Day 1 and 8 of each cycle **Metronomic Schedule**: D1,3,5 every 7 days for 3 weeks of each cycle | 21 days (3 wks) |
| Erlotinib | To be taken whole half hour before breakfast | Dose as appropriate for assigned dose level | Oral | Daily | |

In cases where the patient's BSA > or equal to 2.0, oral Navelbine dose should not exceed twice the dose/$m^2$ (e.g. 120 mg for 60 mg/$m^2$, and 160 mg for 80 mg/$m^2$) per week. In cases where the BSA < or equal to 1.4, these patients will not be recruited into the metronomic arm – this is for patient safety to avoid excessive dose.

The standard "3+3" rule will be employed for dose escalation. Three patients will be accrued at the starting dose levels. If no dose limiting toxicities greater than grade 2 were observed, 3 patients would be entered at the next dose level. If, at any dose level, one of the first 3 patients experiences a DLT, 3 additional patients will be entered at that dose level. If 2 out of 6 patients experience dose-limiting toxicities at this dose level, dose escalation will cease.

The maximally tolerated dose (MTD) will be defined as one dose level below that at which 2 or more patients experienced DLT. If DLT is observed at initial level of either schedule, an additional 3 patients will be accrued at the –1 level. If DLT is observed at this level, the trial will be terminated.

A patient who experiences any DLT will be allowed to continue treatment with a one dose level reduction if the toxicity resolves within 14 days. If this patient experiences a DLT at the lowered dose level, study treatment will be stopped for this patient.

No intra-patient dose escalation will be allowed. Re-entry of a patient accrued at a lower dose into a higher dose cohort will not be allowed. Accrual rate is expected to be 3-4 patients per month. Dose escalation will proceed within each cohort according to the following schedule.

| Number of Patients with DLT at a Given Dose Level | Escalation Decision Rule |
|---|---|
| 0 out of 3 | Enter 3 patients at the next dose level. |
| ≥2 | Dose escalation will be stopped. This dose level will be declared the maximally administered dose (highest dose administered). Three (3) additional patients will be entered at the next lowest dose level if only 3 patients were treated previously at that dose. |
| 1 out of 3 | Enter at least 3 more patients at this dose level.<br>• If 0 of these 3 patients experience DLT, proceed to the next dose level.<br>• If 1 or more of this group suffer DLT, then dose escalation is stopped, and this dose is declared the maximally administered dose. Three (3) additional patients will be entered at the next lowest dose level if only 3 patients were treated previously at that dose. |
| ≤1 out of 6 at highest dose level below the maximally administered dose | This is generally the recommended phase 2 dose. At least 6 patients must be entered at the recommended phase 2 dose. |

### 5.1.1 Oral vinorelbine

Oral ondansetron may be used as prophylactic anti-emetic before administration of vinorelbine on CSV. Full blood counts will be required on Days 1,8,15 of cycle 1 and days 1 and 8 of each subsequent cycle prior to vinorelbine dosing for patients on CSV, and days 1,8, 15 of cycle 1 and day 1 only of every subsequent cycle on MSV. On CSV, for patients who have respond or stable at disease, vinorelbine will be discontinued after 6 cycles. Full blood count is not required for day 8 of cycle 7 and subsequent cycles.

### 5.1.2 Erlotinib

None

## 5.2 Definition of Dose-Limiting Toxicity

All toxicities will be graded according to the National Cancer Institute Common Toxicity Criteria (NCI CTC version 3). In particular, the following toxicities which tend to be more common with vinorelbine use will be recorded in detail: constipation, haematologic toxicities (neutropenia, thrombocytopenia, anemia), neutropenic infections, infections including pneumonia, urinary tract infection, bacteremia, hepatobiliary sepsis; pneumonitis; ileus; hepatitis; fatigue, weight loss, nausea and vomiting.

Hematological Dose Limiting Toxicity (DLT) is defined as follows: grade 4 neutropenia of >7 days duration, neutropenic fever, grade 4 anemia or grade 3-4 thrombocytopenia that occurs during the first cycle of treatment.

Non-hematologic DLT is defined as any grade 3 or grade 4 non-hematologic toxicity that occurs during the first cycle of treatment.

Any toxicity causing a total of 14 days delay will also be considered dose limiting. Toxicities will be classified as related to the study drug unless they were attributable to either underlying tumour progression, concurrent medical condition or a concomitant medication. Any unusual toxicities must be reported to the Principal Investigator

### 5.3 General Concomitant Medication and Supportive Care Guidelines

Because there is a potential for interaction of Vinorelbine and Erlotinib with other concomitantly administered drugs through the cytochrome P450 system, the case report form must capture the concurrent use of all other drugs, over-the-counter medications, or alternative therapies. The Principal Investigator should be alerted if the patient is taking any agent known to affect or with the potential to affect selected CYP450 isoenzymes.

### 5.4 Duration of Therapy

In the absence of treatment delays due to adverse events, treatment may continue indefinitely or until one of the following criteria applies:

- Disease progression,
- Intercurrent illness that prevents further administration of treatment,
- Unacceptable adverse event(s),
- Patient decides to withdraw from the study, or
- General or specific changes in the patient's condition render the patient unacceptable for further treatment in the judgment of the investigator.

### 5.5 Duration of Follow Up

Patients will be followed every 3 monthly for 52 weeks after removal from study or until death, whichever occurs first. Patients removed from study for unacceptable adverse events will be followed until resolution or stabilization of the adverse event.

### 5.6 Criteria for Removal from Study

Patients will be removed from study when any of the criteria listed in Section 5.4 applies. The reason for study removal and the date the patient was removed must be documented in the Case Report Form.

## 6. DOSING DELAYS/DOSE MODIFICATIONS

If patient should encounter significant toxicity in the first cycle of treatment, treatment will be delayed until the recovery of the toxicity. Any delay beyond 2 weeks will result in removal from trial. The subsequent cycle will be started at the next lower dose level (see table).

In addition, the occurrence of a clinically significant toxicity that deemed related to either vinorelbine or erlotinib, in view of safety of the patient, that study drug can be discontinued. Patient will be allowed to continue in the trial with single agent therapy.

| **Dose Level** | **Vinorelbine Dose (Conventional Schedule)** | **Vinorelbine Dose (Metronomic Schedule)** |
|---|---|---|
| -1 | *30 mg/m2, D1,D8 schedule* | *80 mg/week, D1,3,5 every 7 days* |
| 1 | *40 mg/m2, D1,D8 schedule* | *100 mg/week, D1,3,5 every 7 days* |
| 2 | *50 mg/m2, D1,D8 schedule* | *120 mg/week, D1,3,5 every 7 days* |
| 3 | *60 mg/m2, D1,D8 schedule* | *140 mg/week, D1,3,5 every 7 days* |
| 4 | *70 mg/m2, D1,D8 schedule* | *160 mg/week, D1,3,5 every 7 days* |
| 5 | *80 mg/m2, D1,D8 schedule* | *180 mg/week, D1,3,5 every 7 days* |
| **Dose Level** | **Erlotinib Dose** | **Erlotinib Dose** |
| 1 | *100 mg, daily schedule* | *100 mg, daily schedule* |
| 2 | *150 mg, daily schedule* | *150 mg, daily schedule* |

## 7. ADVERSE EVENTS: LIST AND REPORTING REQUIREMENTS

Adverse event (AE) monitoring and reporting is a routine part of every clinical trial.

### 7.1 Adverse Event Characteristics

- **CTCAE term (AE description) and grade:** The descriptions and grading scales found in the revised NCI Common Terminology Criteria for Adverse Events (CTCAE) version 3.0 will be utilized for AE reporting. All appropriate treatment areas should have access to a copy of the CTCAE version 3.0. A copy of the CTCAE version 3.0 can be downloaded from the CTEP web site (http://ctep.cancer.gov).

- **'Expectedness'**: AEs can be 'Unexpected' or 'Expected' (see Section 7.1 above)

for expedited reporting purposes only. 'Expected' AEs (the ASAEL) are ***bold and italicized*** in the CAEPR (Section 7.1.1).

- **Attribution** of the AE:
  - Definite – The AE *is clearly related* to the study treatment.
  - Probable – The AE *is likely related* to the study treatment.
  - Possible – The AE *may be related* to the study treatment.
  - Unlikely – The AE *is doubtfully related* to the study treatment.
  - Unrelated – The AE *is clearly NOT related* to the study treatment.

### 7.2 Expedited Adverse Event Reporting

7.2.1 Expedited AE reporting for this study is required to the IRB.

7.2.2 Serious adverse events has to be reported to the sponsor or regulatory bodies and the Ethics Committee in accordance to the ICH guidelines.

All SAEs that are unexpected and related to the study drug will be reported. The investigator is responsible for informing HSA no later than 15 calendar days after first knowledge that the case qualifies for expedited reporting. Follow-information will be actively sought and submitted as it becomes available. For fatal or life-threatening cases, HSA will be notified as soon as possible but no later than 7 calendar days after first knowledge that a case qualifies, followed by a complete report within 8 additional calendar days.

Withdrawal from the study and therapeutic measures shall be at the discretion of the investigator. All adverse effects, regardless of severity, will be followed by the investigator until satisfactory resolution.

### 7.3 Routine Adverse Event Reporting

All Adverse Events **must** be reported in routine study data submissions.

### 7.4 Secondary AML/MDS

Investigators are required to report cases of secondary AML/MDS occurring on or following treatment to IRB

## 8. PHARMACEUTICAL INFORMATION

A list of the adverse events and potential risks associated with the investigational or commercial agents administered in this study can be found in Section 2.

### 8.1 Oral Vinorelbine

Oral Vinorelbine is manufactured by Pierre-Fabre and supplied locally by Orient

Europharma as NAVELBINE® soft capsules 20 mg and 30 mg.  The capsules also contains the following excipients: ethanol, water - purified, glycerol, macrogol 400, gelatin, sorbitol, sorbitan, medium-chain triglycerides, phosphatidyl choline, glycerides, hypromellose, propylene glycol, edible printing ink E120, titanium dixoide, iron oxide yellow CI77492 and / or iron oxide red CI77491.

Oral vinorelbine has been developed as a line extension of the IV dosage form. An oral dose of 80 mg/m$^2$ was demonstrated to correspond to 30 mg/m$^2$ of the IV formulation and 60 mg/m$^2$ orally to 25 mg/m$^2$ given by the IV route.

**Presentation**

20 mg soft capsule: light brown soft capsule printed N20,
30 mg soft capsule: pink soft capsule printed N30,
Pack size: 1 capsule

**Shelf life**

Store at 2 to 8° C (Refrigerate. Do not freeze) in the original container.
Protect from light.

### 8.2 Erlotinib

The 100 mg and 150 mg strengths are supplied as white film-coated tablets for daily oral administration.

TARCEVA (erlotinib) Tablets, 100 mg: Round, biconvex face and straight sides, white film-coated, printed in gray with "T" and "100" on one side and plain on the other side. Supplied in bottles of 30 tablets (NDC 50242-063-01).

TARCEVA (erlotinib) Tablets, 150 mg: Round, biconvex face and straight sides, white film-coated, printed in maroon with "T" and "150" on one side and plain on the other side. Supplied in bottles of 30 tablets (NDC 50242-064-01).

Store at 25°C (77°F); excursions permitted to 15°-30°C (59°-86°F).

## 9. CORRELATIVE/SPECIAL STUDIES

### 9.1 Rationale for studying pharmacokinetics of oral vinorelbine and erlotinib in relation to CEC/CEP assays as a pharmacodynamic indicators of anti-angiogenesis

Oral vinorelbine is a classic cytotoxic and Erlotinib is a specific oral TK-I of EGFR. Metronomic dosing of chemotherapy using low chronic doses has been suggested as a possible way of inducing an anti-angiogenic effect using classical cytotoxics.

This study will therefore utilize CEC/CEP assays as a pharmacodynamic endpoints to examine both dosing schedules and help determine if metronomic scheduling of vinorelbine has an anti-angiogenic effect. The clinical pharmacokinetics of oral vinorelbine and erlotinib may also have a bearing on the clinical outcome.  The ethnic variability of drug pharmacokinetics is well described for several chemotherapeutic

agents. This is usually related to polymorphic differences across ethnic groups in the way drugs are absorbed, metabolized, distributed and eliminated. Given the paucity of data for this drug and its potential for drug interaction because of its effect of cytochrome p450 enzymes, we therefore propose to study the clinical PK of both drugs in Asian patients in conjunction with a pharmacodynamic study involving the use of CEC/CEP. This will also account for interpatient variability in pharmacokinetics of this drug and allow more accurate interpretation of the pharmacodynamic endpoints. Informed consent for the pharmacokinetic study will be obtained from patients with NSCLC who are planned to start treatment with as in the main study.

### 9.1.1 CEC/CEP assays

Evaluation of CECs and CEPs will be carried out on blood collected at baseline and before each subsequent cycle of treatment (three weeks being defined as one cycle of treatment for both studies) and at time of documented clinical/radiological progression. The endothelial cells will be enumerated using four-colour flow cytometry. Colour-gated Flow Cytometry for the detection of Circulating Endothelial Progenitor Cells (CEPs):

3.5 milliliters of peripheral blood will be collected in ethylenediaminetetraacetic acid (EDTA) tubes through 21G needles. Anti-CD45 will be used to exclude hematopoietic cells; anti-CD31, -CD34, -CD105, -CD106, -CD133, and -P1H[13,14] will be used to enumerate resting and activated circulating endothelial cells (CECs )and circulating endothelial progenitor cells (CEPs) using appropriate analysis gates. Monoclonal antibodies will be conjugated with fluorescein isothiocyanate (FITC), Rphycoerythrin (PE), peridinin chlorophyll protein (PerCP), or allophycocyanin (APC) as appropriate and availability. Cell suspensions after staining will be evaluated by a BD FACS Canto™ II flow cytometer.

Resting CECs is defined as negative for hematopoietic marker CD45; positive for endothelial markers P1H12, CD31, and CD34; negative for activation markers CD105 and CD106; and negative for the progenitor marker CD133.

Activated CECs is defined as CD452, P1H121, CD311, CD341, CD105 or CD1061, and CD1332.

### 9.1.2 Erlotinib and Vinorelbine pharmacokinetics

<u>Vinorelbine</u>

Blood samples (3mL) will be obtained on day 1 and day 10 of dosing at the following time points:

Day 1: 0 hr (pre-dose), 30min, 1hr, 2hr, 4hr, 6hr, 8hr, 24hr, 48hr

Day 10: 0 hr (prior to dosing), 30min, 1hr, 2hr, 4hr, 6hr, 8hr, 24hr

<u>Erlotinib</u>

Erlotinib will commence on day 3 to allow PK profiling of vinorelbine alone.

Pharmacokinetic analysis for erlotinib and its metabolite, OSI-420 will be done on days 3

(after the first dose) and 10 (at steady state).

Day 3: 0hr (predose), 1hr, 2hr, 4hr, 6hr, 8hr, 24hr.

Day 10: Steady state erlotinib pharmacokinetics will be evaluated on day 10 at the following time points: 0 hr (prior to dosing), 1hr, 2hr, 4hr, 6hr, 8hr, 24hr.

A single steady state trough level of erlotinib will be taken on day 22 of every cycle

### 9.1.3 Pharmacogenetics

Blood samples (6 ml) will be obtained at baseline for pharmacogenetic analysis. Genetic polymorphisms of transporter proteins: ABCG2 and MDR1, along with CYP3A5 will be done to determine any correlation with outcome.

Plasma concentration of vinorelbine and erlotinib is determined centrally at Clinical Pharmacology Laboratory, NCCS by previously optimized methods. SNP analysis of the relevant genes of interest will also be analysed in the Clinical Pharmacology Laboratory, NCCS. The patients will be followed in clinic as per main protocol.

## 10. STUDY CALENDAR

Baseline evaluations are to be conducted within 2 weeks prior to start of protocol therapy. Scans and x-rays must be done <u><</u>4 weeks prior to the start of therapy. In the event that the patient's condition is deteriorating, laboratory evaluations should be repeated within 48 hours prior to initiation of the next cycle of therapy.

| | Pre-Study | Wk 1 | Wk 2 | Wk 3 | Wk 4 | Wk 7 | Wk 10 | Wk 13 | Wk 16 | Wk 19 | Wk 22 | >Wk 25 | | Off Study[d] |
|---|---|---|---|---|---|---|---|---|---|---|---|---|---|---|
| Erlotinib | | X--------------------------------------------------------------------X | | | | | | | | | | | | |
| Oral Vinorelbine | | X--------------------------------------------------------------------X | | | | | | | | | | | | |
| Informed consent | X | | | | | | | | | | | | | |
| Demographics | X | | | | | | | | | | | | | |
| Medical history | X | | | | | | | | | | | | | |
| Concurrent meds | X | X--------------------------------------------------------------------X | | | | | | | | | | | | |
| Physical exam | X | X | X | X | X | X | X | X | X | X | X | X[e] | | X |
| Vital signs | X | X | X | X | X | X | X | X | X | X | X | X[e] | | X |
| Height | X | | | | | | | | | | | | | |
| Weight | X | X | | X | | X | | X | | X | | X[e] | | X |
| Performance status | X | X | | X | | X | | X | | X | | X[e] | | X |

| | | | | | | | | | | | | | | |
|---|---|---|---|---|---|---|---|---|---|---|---|---|---|---|
| CBC w/diff, plts | X | X | X | X | X | X | X | X | X | X | X | X[e] | | X |
| Serum chemistry[b] | X | X | X | X | X | X | X | X | X | X | X | X[e] | | X |
| EKG (as indicated) | X | | | | | | | | | | | | | |
| Adverse event evaluation | | X ------------------------------------------------------------------X | | | | | | | | | | | | X |
| Tumor measurements | X | Tumor measurements are repeated every 6 weeks for the first 6 months, then every 12weeks thereafter unless disease progression. Documentation (radiologic) must be provided for patients removed from study for progressive disease. | | | | | | | | | | | | X[d] |
| Radiologic evaluation | X | Radiologic measurements should be performed every 6 weeks for the first 6 months, then every 12 weeks thereafter unless disease progression. | | | | | | | | | | | | X[d] |
| B-HCG | X[c] | | | | | | | | | | | | | |
| Other tests, as appropriate | | | | | | | | | | | | | | |
| Pharmacokinetic studies | **See section 9.0** | | | | | | | | | | | | | |

a: vinorelbine and erlotinib: Dose as assigned; oral.
b: Albumin, alkaline phosphatase, total bilirubin, bicarbonate, BUN, chloride, creatinine, glucose, LDH, potassium, total protein, SGOT[AST], SGPT[ALT], sodium.
c: Serum pregnancy test (women of childbearing potential).
d: Off-study evaluation.
e: For subjects who respond or stable at disease , cycle duration will extend from 3 weekly to 6 weekly after 6 months (8cycles) of treatment.

## 11. MEASUREMENT OF EFFECT

Although response is not the primary endpoint of this trial, patients with measurable disease will be assessed by standard criteria. For the purposes of this study, patients should be re-evaluated every 6 weeks for the first 6 months and subsequently every 12 weeks unless disease progression. In addition to a baseline scan, confirmatory scans will also be obtained 6 weeks following initial documentation of an objective response.
For patients who are discontinued from the study for reasons other than disease progression, tumor evaluation by RECIST will not be necessary once new anti-neoplastic cancer therapy has begun.11.1**Antitumor Effect – Solid Tumors**

Response and progression will be evaluated in this study using the new international criteria proposed by the Response Evaluation Criteria in Solid Tumors (RECIST) Committee [*JNCI* 92(3):205-216, 2000]. Changes in only the largest diameter (unidimensional measurement) of the tumor lesions are used in the RECIST criteria.

#### 11.1.1 **Definitions**

Evaluable for toxicity. All patients will be evaluable for toxicity from the time of their first treatment with Vinorelbine.

Evaluable for objective response. Only those patients who have measurable

disease present at baseline, have received at least one cycle of therapy, and have had their disease re-evaluated will be considered evaluable for response. These patients will have their response classified according to the definitions stated below. (Note: Patients who exhibit objective disease progression prior to the end of cycle 1 will also be considered evaluable.)

### 11.1.2 **Disease Parameters**

Measurable disease. Measurable lesions are defined as those that can be accurately measured in at least one dimension (longest diameter to be recorded) as $\geq$20 mm with conventional techniques (CT, MRI, x-ray) or as $\geq$10 mm with spiral CT scan. All tumor measurements must be recorded in millimeters (or decimal fractions of centimeters).

Non-measurable disease. All other lesions (or sites of disease), including small lesions (longest diameter <20 mm with conventional techniques or <10 mm using spiral CT scan), are considered non-measurable disease. Bone lesions, leptomeningeal disease, ascites, pleural/pericardial effusions, lymphangitis cutis/pulmonis, inflammatory breast disease, abdominal masses (not followed by CT or MRI), and cystic lesions are all non-measurable.

Target lesions. All measurable lesions up to a maximum of 5 lesions per organ and 10 lesions in total, representative of all involved organs, should be identified as **target lesions** and recorded and measured at baseline. Target lesions should be selected on the basis of their size (lesions with the longest diameter) and their suitability for accurate repeated measurements (either by imaging techniques or clinically). A sum of the longest diameter (LD) for all target lesions will be calculated and reported as the baseline sum LD. The baseline sum LD will be used as reference by which to characterize the objective tumor response.

Non-target lesions. All other lesions (or sites of disease) including any measurable lesions over and above the 10 target lesions should be identified as **non-target lesions** and should also be recorded at baseline. Measurements of these lesions are not required, but the presence or absence of each should be noted throughout follow-up.

### 11.1.3 **Methods for Evaluation of Measurable Disease**

All measurements should be taken and recorded in metric notation using a ruler or calipers. All baseline evaluations should be performed as closely as possible to the beginning of treatment and never more than 4 weeks before the beginning of the treatment.

The same method of assessment and the same technique should be used to characterize each identified and reported lesion at baseline and during follow-up.

### 11.1.4 **Response Criteria**

11.1.4.1 **Evaluation of Target Lesions**

| | |
|---|---|
| Complete Response (CR): | Disappearance of all target lesions |
| Partial Response (PR): | At least a 30% decrease in the sum of the longest diameter (LD) of target lesions, taking as reference the baseline sum LD |
| Progressive Disease (PD): | At least a 20% increase in the sum of the LD of target lesions, taking as reference the smallest sum LD recorded since the treatment started or the appearance of one or more new lesions |
| Stable Disease (SD): | Neither sufficient shrinkage to qualify for PR nor sufficient increase to qualify for PD, taking as reference the smallest sum LD since the treatment started |

11.1.4.2 **Evaluation of Non-Target Lesions**

| | |
|---|---|
| Complete Response (CR): | Disappearance of all non-target lesions and normalization of tumor marker level |
| Incomplete Response/ Stable Disease (SD): | Persistence of one or more non-target lesion(s) and/or maintenance of tumor marker level above the normal limits |
| Progressive Disease (PD): | Appearance of one or more new lesions and/or unequivocal progression of existing non-target lesions |

Although a clear progression of "non-target" lesions only is exceptional, the opinion of the treating physician should prevail in such circumstances, and the progression status should be confirmed at a later time by the review panel (or Principal Investigator).

11.1.4.3 **Evaluation of Best Overall Response**

The best overall response is the best response recorded from the start of the treatment until disease progression/recurrence (taking as reference for progressive disease the smallest measurements recorded since the treatment started). The patient's best response assignment will depend on the achievement of both measurement and confirmation criteria.

| Target Lesions | Non-Target Lesions | New Lesions | Overall Response | Best Response for this Category Also Requires: |
|---|---|---|---|---|
| CR | CR | No | CR | ≥4 wks. confirmation |
| CR | Non-CR/Non-PD | No | PR | ≥4 wks. confirmation |
| PR | Non-PD | No | PR | |
| SD | Non-PD | No | SD | documented at least once ≥4 wks. from baseline |
| PD | Any | Yes or No | PD | no prior SD, PR or CR |
| Any | PD* | Yes or No | PD | |
| Any | Any | Yes | PD | |

* In exceptional circumstances, unequivocal progression in non-target lesions may be accepted as disease progression.

Note: Patients with a global deterioration of health status requiring discontinuation of treatment without objective evidence of disease progression at that time should be reported as "*symptomatic deterioration*". Every effort should be made to document the objective progression even after discontinuation of treatment.

A Clinical Benefit Rate (CBR) consisting of the total number of CR+PR+SD will be used as a secondary endpoint.

### 11.1.5 Duration of Response

Duration of overall response: The duration of overall response is measured from the time measurement criteria are met for CR or PR (whichever is first recorded) until the first date that recurrent or progressive disease is objectively documented (taking as reference for progressive disease the smallest measurements recorded since the treatment started).

The duration of overall CR is measured from the time measurement criteria are first met for CR until the first date that recurrent disease is objectively documented.

Duration of stable disease: Stable disease is measured from the start of the treatment until the criteria for progression are met, taking as reference the smallest measurements recorded since the treatment started.

### 11.1.6 Overall Survival

The duration of overall survival will be calculated from the date of registration to the date of death.

## 12. DATA REPORTING / REGULATORY REQUIREMENTS

### 12.1 Definition of Adverse Events

Definition of an adverse event: is any undesirable event associated with the use of a drug, whether or not considered drug related, and includes any side effect, injury, toxicity or sensitivity reactions. It also includes any undesirable clinical or laboratory event which is not normally observed in the patient.

## 12.2 Reporting of Adverse Events

Adverse events will be collected after the patient has been enrolled. If a patient experiences an adverse event after the informed consent document is signed but prior to assignment to treatment, the event will NOT be reported unless the investigator feels that the event may have been caused by a protocol procedure.

Prior to enrollment, study site personnel will note the occurrence and nature of each patient's medical condition(s). During the study, site personnel will again note any change in the condition(s) and /or the occurrence and nature of any adverse events.

Patients will be monitored closely for adverse events while receiving study agent and for 30 days after last dose of study agent in order to detect delayed toxicity. After this period, investigators should only report serious adverse events that are felt to be causally related to study agent or to protocol procedure.

The NCI CTC scale will be used as an aid for listing adverse events. All adverse events will be reported in Case Report Forms of the study.

## 12.3 Definition of Serious Adverse Events

Definition of a serious adverse event: is any event that is fatal, life-threatening, requires or prolongs hospitalisation, results in persistent or significant disability or incapacity, a congenital anomaly or birth defect, an important medical event. Important medical events are those which may not be immediately life-threatening, but are of major clinical significance. They may jeopardise the subject and may require intervention to prevent one of the other serious outcomes.

## 12.4 Expedited Reporting Guidelines

Serious adverse events have to be reported to the sponsor or regulatory bodies and the Ethics Committee in accordance to the ICH guidelines.

# 13. STATISTICAL CONSIDERATIONS

This is a phase I dose-finding study. All patients enrolled in this study will be evaluated for toxicity and response.

13.1 Analysis

• The primary aim of this study is to determine the dose limiting toxicities of oral vinorelbine combined with erlotinib and the maximal tolerated dose (MTD) of this drug combination in patients with non-small cell lung cancer. The toxicity profile will be graded as described above and reported with the corresponding dose level.

• The MTD will be dose at which less than 2 DLTs occur. Steady state plasma drug

concentrations, area under the curve values, volume of distribution and clearance of oral vinorelbine and erlotinib in each patient will be calculated and correlated with dose. These quantities will be summarized for all patients.

• Response rates (Complete response, partial response, stable disease) to study treatment will be described based on RECIST criteria and summarized by dose. Overall survival will be measured from registration to death and analyzed by the Kaplan-Meier method.

• Exploratory analysis of known functional SNPs in the transporter genes will be described in relation to dose and response. An exploratory analysis of the changes on CEC/CEP assessment of angiogenesis in each group would be performed.

13.2 Sample size considerations:

As this is a phase 1 study, no formal sample size calculation is possible. The number of patients accrued will depend on the DLT reached on each arm of the study. Between 3-6 patients will be observed on each dose level~~s~~ and an expanded cohort of 6 patients will be observed on the MTD. The anticipated accrual to this study is between 12 (if there are at least 2 DLT at dose level 1 and DLT at level -1) to 78 patients (if dose level 5 is reached and the cohort receiving erlotinib 150 mg is included).

## REFERENCES


1. Shepherd FA, Rodrigues Pereira J, Ciuleanu T, Tan EH, et al. Erlotinib in previously treated non-small-cell lung cancer. N Engl J Med. 2005 Jul 14;353(2):123-32.

2. Shepherd FA, Dancy J, Ramlau R, et al. Prospective randomized trial of docetaxel versus best supportive care in patients with non-small-cell lung cancer previously treated with platinum-based chemotherapy. J Clin Oncol. 2000 May;18(10):2095-103.

3. Hanna N, Shepherd FA, Fossella FV, et al. Randomized phase III trial of pemetrexed versus docetaxel in patients with non-small-cell lung cancer previously treated with chemotherapy. J Clin Oncol. 2004 May 1;22(9):1589-97.

4. Fossella FV, DeVore R, Kerr RN, et al. Randomized phase III trial of docetaxel versus vinorelbine or ifosfamide in patients with advanced non-small-cell lung cancer previously treated with platinum-containing chemotherapy regimens. The TAX 320 Non-Small Cell Lung Cancer Study Group. J Clin Oncol. 2000 Jun;18(12):2354-62

5. Erjala K, Raitanen M, Kulmala J et al. Concurrent use of vinorelbine and gefitinib induces supra-additive effect in head and neck squamous cell carcinoma cell lines. J Cancer Res Clin Oncol. 2007 Mar;133(3):169-76J Cancer Res Clin Oncol. 2007 Mar;133(3):169-76

6. Chen YM, Liu JM, Chou TY, et al. Phase II randomized study of daily gefitinib

treatment alone or with vinorelbine every 2 weeks in patients with adenocarcinoma of the lung who failed at least 2 regimens of chemotherapy. Cancer. 2007 May 1;109(9):1821-8.

7. Bocci G, Nicolaou KC, Kerbel RS. Protracted low-dose effects on human endothelial cell proliferation and survival in vitro reveal a selective antiangiogenic window for various chemotherapeutic drugs. Cancer Res. 2002 Dec 1;62(23):6938-43.

8. Bertolini F, Paul S, Mancuso P, et al. Maximum tolerable dose and low-dose metronomic chemotherapy have opposite effects on the mobilization and viability of circulating endothelial progenitor cells.Cancer Res 2003; 63: 4342-6

9. Browder T, Butterfield CE, Kräling BM, et al. Antiangiogenic scheduling of chemotherapy improves efficacy against experimental drug-resistant cancer. Cancer Res 2000; 60: 1878-86

10. Klement G, Huang P, Mayer B, et al. Differences in therapeutic indexes of combination metronomic chemotherapy and an anti-VEGFR-2 antibody in multidrug-resistant human breast cancer xenografts. Clin Cancer Res 2002; 8: 221-32

11. L. Rajdev, Q. Dai, G. Goldberg, S. Hoschander et al. A phase 1 study of oral navelbine in patients with advanced solid tumors. J Clin Oncol, 2006 ASCO Annual Meeting Proceedings Part I. Vol 24, No. 18S (June 20 Supplement), 2006: 13095

12. Shaked Y, Emmenegger U, Francia G, et al. Low-dose metronomic combined with intermittent bolus-dose cyclophosphamide is an effective long-term chemotherapy treatment strategy. Cancer Res. 2005;65(16):7045-51.

13. Solovey A, Lin Y, Browne P, Choong S, Wayner E, Hebbel RP. Circulating activated endothelial cells in sickle cell anemia. N Engl J Med; 337(22):1584-90, 1997.

14. St Croix B, Rago C, Velculescu V, Traverso G, Romans KE, Montgomery E, Lal A, Riggins GJ, Lengauer C, Vogelstein B, Kinzler KW. Genes expressed in human tumor endothelium. Science; 289(5482):1197-202, 2000.

## APPENDIX A

### Performance Status Criteria

| **ECOG Performance Status Scale** | | **Karnofsky Performance Scale** | |
|---|---|---|---|
| Grade | Descriptions | Percent | Description |
| 0 | Normal activity. Fully active, able to carry on all pre-disease performance without restriction. | 100 | Normal, no complaints, no evidence of disease. |
| | | 90 | Able to carry on normal activity; minor signs or symptoms of disease. |
| 1 | Symptoms, but ambulatory. Restricted in physically strenuous activity, but ambulatory and able to carry out work of a light or sedentary nature (e.g., light housework, office work). | 80 | Normal activity with effort; some signs or symptoms of disease. |
| | | 70 | Cares for self, unable to carry on normal activity or to do active work. |
| 2 | In bed <50% of the time. Ambulatory and capable of all self-care, but unable to carry out any work activities. Up and about more than 50% of waking hours. | 60 | Requires occasional assistance, but is able to care for most of his/her needs. |
| | | 50 | Requires considerable assistance and frequent medical care. |
| 3 | In bed >50% of the time. Capable of only limited self-care, confined to bed or chair more than 50% of waking hours. | 40 | Disabled, requires special care and assistance. |
| | | 30 | Severely disabled, hospitalization indicated. Death not imminent. |
| 4 | 100% bedridden. Completely disabled. Cannot carry on any self-care. Totally confined to bed or chair. | 20 | Very sick, hospitalization indicated. Death not imminent. |
| | | 10 | Moribund, fatal processes progressing rapidly. |
| 5 | Dead. | 0 | Dead. |
