# Supplementary material for: Phase I Study of Oral Vinorelbine in Combination with Erlotinib in Advanced Non-Small Cell Lung Cancer (NSCLC) Using Two Different Schedules
Source: PLoS One. 2016 May 2;11(5):e0154316. doi: 10.1371/journal.pone.0154316 (PMC4852941; doi:10.1371/journal.pone.0154316)
Supplement: S1 Table — (DOCX) [file pone.0154316.s003.docx]

**S1 Table. Adverse events in all cycles of treatment for patients in each dose level of the CSV group**

| Adverse Event | CSV  Dose Level 1 (N=3) | | | CSV  Dose Level 2 (N=3) | | | CSV  Dose Level 3 (N=3) | | CSV  Dose Level 4 (N=3) | | CSV  Dose Level 5 (N=3) | | CSV  Dose Level 6 (N=1) | | |
| --- | --- | --- | --- | --- | --- | --- | --- | --- | --- | --- | --- | --- | --- | --- | --- |
|  | **All grades, n (%)** | | **Grades 3/4, n (%)** | | **All grades, n (%)** | **Grades 3/4, n (%)** | **All grades, n (%)** | **Grades 3/4, n (%)** | **All grades, n (%)** | **Grades 3/4, n (%)** | **All grades, n (%)** | **Grades 3/4, n (%)** | **All grades, n (%)** | | **Grades 3/4, n (%)** |
| Gastrointestinal |  |  | |  | |  |  |  |  |  |  |  |  |  | |
| Diarrhea | 1 (33) | 0 (0) | | 1 (33) | | 0 (0) | 2 (67) | 0 (0) | 3 (100) | 0 (0) | 3 (100) | 0 (0) | 0 (0) | 0 (0) | |
| Nausea | 0 (0) | 0 (0) | | 0 (0) | | 0 (0) | 0 (0) | 0 (0) | 2 (67) | 0 (0) | 1 (33) | 0 (0) | 0 (0) | 0 (0) | |
| Vomiting | 0 (0) | 0 (0) | | 1 (33) | | 0 (0) | 0 (0) | 0 (0) | 2 (67) | 0 (0) | 1 (33) | 0 (0) | 0 (0) | 0 (0) | |
| Constipation | 0 (0) | 0 (0) | | 1 (33) | | 0 (0) | 0 (0) | 0 (0) | 1 (33) | 0 (0) | 2 (67) | 0 (0) | 1 (100) | 0 (0) | |
| Dysphagia/ heartburn | 0 (0) | 0 (0) | | 0 (0) | | 0 (0) | 0 (0) | 0 (0) | 1 (33) | 0 (0) | 1 (33) | 0 (0) | 0 (0) | 0 (0) | |
| Stomatitis | 1 (33) | 0 (0) | | 2 (67) | | 0 (0) | 0 (0) | 0 (0) | 1 (33) | 0 (0) | 2 (67) | 0 (0) | 0 (0) | 0 (0) | |
| Anorexia/loss of appetite | 0 (0) | 0 (0) | | 0 (0) | | 0 (0) | 0 (0) | 0 (0) | 0 (0) | 0 (0) | 0 (0) | 0 (0) | 0 (0) | 0 (0) | |
| Dermatological |  |  | |  | |  |  |  |  |  |  |  |  |  | |
| Rash | 3 (100) | 0 (0) | | 3 (100) | | 0 (0) | 3 (100) | 0 (0) | 3 (100) | 0 (0) | 3 (100) | 0 (0) | 0 (0) | 0 (0) | |
| Pruritus | 3 (100) | 0 (0) | | 3 (100) | | 0 (0) | 2 (67) | 0 (0) | 3 (100) | 0 (0) | 3 (100) | 0 (0) | 0 (0) | 0 (0) | |
| Dry skin | 3 (100) | 0 (0) | | 3 (100) | | 0 (0) | 3 (100) | 0 (0) | 3 (100) | 0 (0) | 3 (100) | 0 (0) | 1 (100) | 0 (0) | |
| Alopecia | 0 (0) | 0 (0) | | 0 (0) | | 0 (0) | 0 (0) | 0 (0) | 0 (0) | 0 (0) | 0 (0) | 0 (0) | 0 (0) | 0 (0) | |
| Metabolic |  |  | |  | |  |  |  |  |  |  |  |  |  | |
| Hyponatremia | 0 (0) | 0 (0) | | 0 (0) | | 0 (0) | 0 (0) | 0 (0) | 0 (0) | 0 (0) | 0 (0) | 0 (0) | 1 (100) | 1 (100) | |
| Hematologic/Infection |  |  | |  | |  |  |  |  |  |  |  |  |  | |
| Anemia | 0 (0) | 0 (0) | | 0 (0) | | 0 (0) | 0 (0) | 0 (0) | 0 (0) | 0 (0) | 0 (0) | 0 (0) | 0 (0) | 0 (0) | |
| Non-febrile neutropenia | 0 (0) | 0 (0) | | 0 (0) | | 0 (0) | 0 (0) | 0 (0) | 1 (33) | 1 (33) | 1 (33) | 1 (33) | 0 (0) | 0 (0) | |
| Infection with normal ANC | 0 (0) | 0 (0) | | 0 (0) | | 0 (0) | 0 (0) | 0 (0) | 0 (0) | 0 (0) | 0 (0) | 0 (0) | 0 (0) | 0 (0) | |
| Neutropenic fever | 0 (0) | 0 (0) | | 0 (0) | | 0 (0) | 0 (0) | 0 (0) | 0 (0) | 0 (0) | 0 (0) | 0 (0) | 0 (0) | 0 (0) | |
| Pain |  |  | |  | |  |  |  |  |  |  |  |  |  | |
| Nose | 0 (0) | 0 (0) | | 1 (33) | | 0 (0) | 0 (0) | 0 (0) | 0 (0) | 0 (0) | 0 (0) | 0 (0) | 0 (0) | 0 (0) | |
| Face | 0 (0) | 0 (0) | | 0 (0) | | 0 (0) | 0 (0) | 0 (0) | 1 (33) | 0 (0) | 0 (0) | 0 (0) | 0 (0) | 0 (0) | |
| Joint | 0 (0) | 0 (0) | | 0 (0) | | 0 (0) | 0 (0) | 0 (0) | 0 (0) | 0 (0) | 1 (33) | 0 (0) | 0 (0) | 0 (0) | |
| Muscle | 0 (0) | 0 (0) | | 0 (0) | | 0 (0) | 0 (0) | 0 (0) | 0 (0) | 0 (0) | 0 (0) | 0 (0) | 0 (0) | 0 (0) | |
| Nail | 0 (0) | 0 (0) | | 0 (0) | | 0 (0) | 0 (0) | 0 (0) | 0 (0) | 0 (0) | 0 (0) | 0 (0) | 0 (0) | 0 (0) | |
| Others |  |  | |  | |  |  |  |  |  |  |  |  |  | |
| Dry eyes | 0 (0) | 0 (0) | | 0 (0) | | 0 (0) | 0 (0) | 0 (0) | 0 (0) | 0 (0) | 0 (0) | 0 (0) | 0 (0) | 0 (0) | |
| Keratitis | 0 (0) | 0 (0) | | 0 (0) | | 0 (0) | 0 (0) | 0 (0) | 0 (0) | 0 (0) | 0 (0) | 0 (0) | 0 (0) | 0 (0) | |
| Fatigue | 1 (33) | 0 (0) | | 1 (0) | | 0 (0) | 0 (0) | 0 (0) | 1 (33) | 0 (0) | 2 (67) | 0 (0) | 0 (0) | 0 (0) | |
| Giddiness | 0 (0) | 0 (0) | | 0 (0) | | 0 (0) | 0 (0) | 0 (0) | 1 (33) | 0 (0) | 0 (0) | 0 (0) | 0 (0) | 0 (0) | |
| Neuropathy | 1 (33) | 0 (0) | | 0 (0) | | 0 (0) | 0 (0) | 0 (0) | 0 (0) | 0 (0) | 2 (67) | 0 (0) | 1 (100) | 0 (0) | |
| Paronychia | 0 (0) | 0 (0) | | 0 (0) | | 0 (0) | 0 (0) | 0 (0) | 1 (33) | 0 (0) | 1 (33) | 0 (0) | 0 (0) | 0 (0) | |
